# CBP mediated DOT1L acetylation confers DOT1L stability and promotes cancer metastasis

**DOI:** 10.7150/thno.39013

**Published:** 2020-01-01

**Authors:** Chaohua Liu, Qiaoyan Yang, Qian Zhu, Xiaopeng Lu, Meiting Li, Tianyun Hou, Zhiming Li, Ming Tang, Yinglu Li, Hui Wang, Yang Yang, Haiying Wang, Ying Zhao, He Wen, Xiangyu Liu, Zebin Mao, Wei-Guo Zhu

**Affiliations:** 1 Key Laboratory of Carcinogenesis and Translational Research, Ministry of Education, Beijing Key Laboratory of Protein Posttranslational Modifications and Cell Function, Department of Biochemistry and Molecular Biology, School of Basic Medical Sciences, Peking University Health Science Center, Beijing 100191, China; 2 Guangdong Key Laboratory for Genome Stability and Human Disease Prevention, Department of Biochemistry and Molecular Biology, School of Medicine, Shenzhen University, Shenzhen 518060, China

**Keywords:** DOT1L, CBP, Acetylation, Degradation, Metastasis

## Abstract

**Background and Aim**: DOT1L regulates various genes involved in cancer onset and progression by catalyzing H3K79 methylation, but how DOT1L activity itself is regulated is unclear. Here, we aimed to identify specific DOT1L post-translational modifications that might regulate DOT1L activity and thus impact on colorectal cancer (CRC) progression.

**Methods**: We conducted affinity purification and mass spectrometry to explore DOT1L post-translational modifications. We then established transwell migration and invasion assays to specifically investigate the role of DOT1L(K358) acetylation on CRC cellular behavior *in vitro* and a bioluminescence imaging approach to determine the role of DOT1L(K358) acetylation in CRC metastasis *in vivo*. We performed chromatin immunoprecipitation to identify DOT1L acetylation-controlled target genes. Finally, we used immunohistochemical staining of human tissue arrays to examine the relevance of DOT1L(K358) acetylation in CRC progression and metastasis and the correlation between DOT1L acetylation and CBP.

**Results**: We found that CBP mediates DOT1L K358 acetylation in human colon cancer cells and positively correlates with CRC stages. Mechanistically, DOT1L acetylation confers DOT1L stability by preventing the binding of RNF8 to DOT1L and subsequent proteasomal degradation, but does not affect its enzyme activity. Once stabilized, DOT1L can catalyze the H3K79 methylation of genes involved in epithelial-mesenchymal transition, including *SNAIL* and *ZEB1*. An acetylation mimic DOT1L mutant (Q358) could induce a cancer-like phenotype *in vitro,* characterized by metastasis and invasion. Finally, DOT1L(K358) acetylation correlated with CRC progression and a poor survival rate as well as with high CBP expression.

**Conclusions**: DOT1L acetylation by CBP drives CRC progression and metastasis. Targeting DOT1L deacetylation signaling is a potential therapeutic strategy for DOT1L-driven cancers.

## Introduction

Colorectal cancer (CRC) is the third most common malignant neoplasm and a leading cause of death worldwide 1. CRC is typically diagnosed after tumor invasion and metastasis has occurred, which greatly limits patient survival outcomes: half of all CRC patients develop distant metastases and the 5-year survival rate of patients with metastatic CRC is <20% 2-5. Dysregulated epigenetic regulatory processes in CRC, particularly aberrant histone methylation mediated by histone methyltransferases (HMTs), have drawn increasing interest in recent years. For example, the HMT DOT1L is highly expressed in CRC, where it acts as an oncoprotein and is associated with poor survival and CRC aggressiveness 6, 7. DOT1L expression also correlates with cancer stemness and tumorigenesis 8, 9. Improving our understanding the DOT1L regulatory mechanisms in the context of CRC might help guide new therapeutic approaches.

We already know that DOT1L/KMT4 is a non-SET domain methyltransferase that catalyzes H3K79 methylation 10-12 and has crucial roles in diverse cellular processes, including development, differentiation and proliferation 13-15. In the cancer context, several studies have implicated a role for DOT1L in the development of leukemia and solid tumors 14, 16, 17, including CRC 6. Here, DOT1L promotes CRC stemness and tumorigenesis by inducing H3K79 methylation 6, but the underlying DOT1L target genes driving oncogenesis and CRC progression is unclear.* SNAIL* and *ZEB1* are important transcription factors that regulate the epithelial-mesenchymal transition (EMT) and are associated with cancer-cell metastasis and invasion 18-20. When DOT1L is highly expressed, H3K79 methylation levels around the *SNAIL* and *ZEB1* promoters are high; this effect leads to *SNAIL* and *ZEB1* expression and repressed *CDH1* (encoding E-cadherin) transcription 21. Thus, the balance of DOT1L levels is crucial for regulating H3K79 methylation, *SNAIL* and *ZEB1* expression, and eventual changes in cell metastasis and invasion. Although KDM2B may act as a histone demethylase for H3K79me2/3 has been reported 22, its mechanism is not extensively applied in the research about oncogenesis and cell metastasis.

Several mechanisms have been proposed to explain how DOT1L is regulated, but most of these have focused on how protein-protein interactions mediate DOT1L activity 23-25. A recent study showed that DOT1L levels decrease in U2OS cells during the early DNA damage response 26, suggesting that DOT1L could be regulated in protein levels. How DOT1L stability is regulated, however, has not been explored. A key mechanism by which cellular protein levels are maintained is degradation *via* the proteasome pathway 27-29. A pre-requisite to proteasomal degradation is usually post-translational modification (PTM), such as acetylation or ubiquitination 30, 31. In terms of acetylation, predominant acetyltransferases include p300, CBP, PCAF and Tip60, which all catalyze histone and non-histone acetylation 32, 33. Conversely, histone deacetylases (HDACs) remove acetyl groups from proteins to affect protein activity or stability 34-36. Together, histone acetyltransferases and HDACs control the acetylation status of targeted proteins, leading to changes in the targeted protein activity or stability.

Given the anticancer potential of DOT1L, DOT1L inhibitors (such as pinometostat) have been designed to target the DOT1L S-adenosyl-l-methionine (SAM) binding pocket to inhibit H3K79 methylation 37. The low sensitivity of these inhibitors, however, have limited their clinical benefit for patients, and the optimal dose is unclear 38. Here we aimed to identify factors that influence DOT1L stability and can thus affect H3K79 methylation levels in CRC. We identified a unique DOT1L regulator, which might serve as a potential target for combating hyper-expressed DOT1L-driven tumors.

## Materials and methods

### Cell culture

All cell lines used in this study were obtained from the American Type Culture Collection (ATCC). HCT116 cells were grown in McCoy's 5A with 10% (vol/vol) fetal bovine serum (FBS) and the appropriate amount of penicillin/streptomycin (penicillin, 100 IU/ml; streptomycin, 100 μg/ml); LoVo, HT-29, SW480, SW116 cells were grown in Dulbecco's modified Eagle's medium (DMEM) supplemented with 10% (vol/vol) FBS and the appropriate amount of penicillin/streptomycin (penicillin, 100 IU/ml; streptomycin, 100 μg/ml); DLD-1 cells were grown in RPMI1640 medium with 10% (vol/vol) FBS and the appropriate amount of penicillin/streptomycin (penicillin, 100 IU/ml; streptomycin, 100 μg/ml). All cells were maintained in a humidified incubator equilibrated with 5% CO_2_ at 37°C. CCD841 cells were cultured in L-15 medium supplemented with 10% FBS and without CO_2_.

### Antibodies

The antibodies in this study included: H3K79me1 (ab2886, Abcam), H3K79me2 (ab3594, Abcam), H3K79me3 (ab2621, Abcam), Histone H3 (ab1971, Abcam), β-actin (TA-09, Zsbio), DOT1L (A300-953A, Bethyl; sc-390879, Santa Cruz), Acetylated-Lysine (9441S, Cell Signaling Technology), DOT1L-Ac-K358 (PTM BIO Inc), Flag (F1804, Sigma), CBP (sc-369, Santa Cruz), p300 (sc-585, Santa Cruz), E-cadherin (3195S, Cell Signaling Technology and 610404, BD Biosciences), Snail (sc-28199, Santa Cruz), ZEB1 (sc-25388, Santa Cruz), α-tubulin (sc-3908103, Santa Cruz), glutathione S-transferase (C1303, Applygen), green fluorescent protein (GFP) (D153-3, MBL) and His (PM032, MBL).

### Plasmids

Full-length DOT1L or various fragments (N-terminal domain, 1-416aa; N-terminal central region domain, 417-579aa; STAT1 binding domain, 580-1138aa; C-terminal domain, 1139-1537aa) were cloned into pGEX-4T-3 vectors (Addgene, USA). Site-specific DOT1L mutations (K358Q, K358R, K1228R) were generated using a site-directed mutagenesis kit (Vazyme, China). The pHBLV-luci control vector was purchased from Hanbio Biotechnology, China. DOT1L(WT), DOT1L(K358Q) and DOT1L(K358R) were cloned into a pHBLV-luci control vector. Transient and stable transfections of these plasmids were performed using Lipofectamine 2000 (Invitrogen, USA), according to the manufacturer's protocol.

### Mass spectrometry

Cellular extracts of SW116 and HCT116 cells or Flag-DOT1L-transfected HCT116 cells were subjected to immunoprecipitation with anti-DOT1L or anti-Flag antibodies. The endogenous DOT1L immune-precipitates or Flag-DOT1L immunoprecipitates were separated by SDS-PAGE and excised after staining with coomassie brilliant blue (CBB).

Each gel band was excised and digested in-gel with 2-10 ng/μL sequencing grade trypsin in 50 mM ammonium bicarbonate overnight at 37 °C. Prior to the addition of the enzyme, gel pieces were dehydrated in acetonitrile, incubated in 10 mM DTT in 50 mM ammonium bicarbonate at 56 ºC for 40 min, incubated in 55 mM iodoacetamide in 50 mM ammonium biocarbonate at ambient temperature for 1 h in the dark, then dehydrated again. The resulting peptides were extracted twice with 5% formic acid/50% acetonitrile, then vacuum-centrifuged to dryness.

For LC-MS/MS analysis, the samples were reconstituted in 0.2% formic acid, loaded onto a 100 μm × 2 cm pre-column and separated on a 75 μm × 15 cm capillary column with a laser-pulled sprayer. Both columns were packed in-house with 4 μm C18 bulk materials (InnosepBio, P.R.China). An Easy nLC 1000 system (Thermo Scientific, USA) was used to deliver the following HPLC gradient: 5-30% B in 60 min, 30-75% B in 4 min, then held at 75% B for 20 min (A = 0.1% formic acid in water, B = 0.1% formic acid in acetonitrile). The eluted peptides were sprayed into a Velos Pro Orbitrap Elite mass spectrometer (Thermo Scientific, USA) equipped with a nano-ESI source. The mass spectrometer was operated in data-dependent mode with a full MS scan in FT mode at a resolution of 120,000 followed by CID (Collision Induced Dissociation) MS/MS scans on the 15 most abundant ions in the initial MS scan (the Analytical Instrumentation Center of Peking University (PKUAIC)). Two replicate experiments were conducted. The raw data files were converted to mascot generic format (“.mgf”) using MSConvert before database searching. Mascot (version 2.3.02) was used for the database search with the following parameters: Carbamidomethyl (Cys) as fixed modification, Oxidation (Met), Acetylation (Lys) and Ubiquitination (Lys) as variable modification; +/- 10 ppm for peptide pass tolerance and +/- 0.6 Da for fragment mass tolerance; max missed cleavages 2.

### Dot blot assay

Increasing concentrations of the indicated peptides were immobilized on nitrocellulose membranes. The membranes were dried using a vacuum pump and subjected to western blotting with the indicated antibodies. P1 refers to acetyl-DOT1L(K358) peptide 1 (NAATPT-(acetyl)K-GPEGKC); P2 refers to acetyl-DOT1L(K358) peptide 2 (CATPT-(acetyl)K-GPEGKVA); P3 refers to unmodified-DOT1L(K358) peptide 3 (CNAATPTKGPEGKVA).

### DOT1L protein turnover

The turnover rate of endogenous DOT1L in HCT116 cells was determined using cycloheximide (CHX) (01810; Sigma-Aldrich) to inhibit protein synthesis. HCT116 cells were transiently transfected with DOT1L(WT), DOT1L(K358Q) or DOT1L(K358R), and CHX was added to the culture media 36 h after transfection to a final concentration of 20 μg/ml. The cells were harvested at the indicated time points, and equal amounts of the cell lysates were subjected to SDS-PAGE and analysed by western blotting.

### Chromatin Protein Extraction

Cells (1-3×10^6^) were lysed on ice in buffer I (150 mM NaCl, 50 mM Hepes, pH 7.5, 1 mM EDTA), with 0.1% Triton X-100 and a protease inhibitor cocktail (Roche) for 3 min. After centrifugation at 13,000*g* for 3 min, the detergent extractable (Dt) supernatant was collected. The cell pellets were then washed twice in PBS and lysed in buffer II (150 mM NaCl, 50 mM Hepes pH 7.5, 1 mM EDTA, 200 μg/ml RNaseA) with a protease inhibitor cocktail for 30 min at 25°C with gentle agitation. After centrifugation at 12,000*g* for 3 min, the remaining pellets (chromatin protein) were re-suspended in 2×SDS loading buffer, boiled, and sonicated for solubilization.

### GST pull-down assay

GST or GST-tagged plasmids were transformed in *E. coli* BL21 cells (TianGen) and induced using 0.1 mM IPTG (Isopropyl β-D-1-thiogalactopyranoside) at 16°C overnight. The GST or GST-fusion proteins were purified using glutathione-Sepharose 4B beads (GE Healthcare, Kings Park, NY, USA) and then washed with TEN buffer [20 mM Tris-HCl (pH7.4), 0.1 mM EDTA, and 100 mM NaCl]. For GST pull down, recombinant proteins or cell lysates were separately incubated overnight with GST fusion proteins at 4°C. The beads were washed three times with TEN buffer and then boiled with 2×SDS loading buffer before analysis by SDS-PAGE and western blotting with the indicated antibodies.

### Reverse transcription and quantitative real-time PCR (qRT-PCR)

Total RNA was isolated with TRIzol reagent (TianGen, Beijing, China). cDNA was synthesized from 2 μg RNA using a Quant script RT Kit (Promega, WI, USA), according to the manufacturer's instructions. Quantitation of all gene transcripts was performed by qRT-PCR using Power SYBR Green PCR Master Mix and an ABI PRISM 7500 sequence detection system (Applied Biosystems, Foster City, CA). The following specific primers were used: *GADPH*, 5′-GAAGGTGAAGGTCGGAGTC-3′ and 5′-GAAGATGGTGATGGGATTTC-3′; *DOT1L*, 5′-CTGCCGGTCTACGATAAACATC-3′ and 5′-AGCTTGAGATCCGGGATTTCT-3′; *SNAIL*, 5′-AGCCTGGGTGCCCTCAAGATG-3′ and 5′-CTTGGTGCTTGTGGAGCAGGGAC-3′; *ZEB1*, 5′-GCACCTGAAGAGGACCAGAG-3′ and 5′-GTGTAACTGCACAGGGAGCA-3′. Relative expression was calculated according to the ΔCt method with normalization to *GADPH*.

### RNAi assay

RNAi oligonucleotides were transfected into cells using Lipofectamine 2000 (Invitrogen), according to the manufacturer's instructions. Cells were harvested 48-72 h after transfection and the lysates were analyzed by western blotting. The RNAi oligonucleotide sequences were as follows: DOT1L siRNA#1, 5′-GCUGGAGCUGAGACUGAAG-3′, DOT1L siRNA#2, 5′-GCUCGCUAUGGAGAAUUAC-3′; RNF8 siRNA#1, 5′-CCAAAGAATGACCAAATGATA-3′, RNF8 siRNA#2, 5′-TGGAGCAACTAGAGAAGACTT-3′; RNF168 siRNA#1, 5′- GGCGAAGAGCGAUGGAAGA-3′, RNF168 siRNA#2, 5′-CGTGGAACTGTGGACGATAATTCAA-3′; CBP siRNA#1, 5′-GGCCUCCUCAAUAGUAACUTT-3′, CBP siRNA#2, 5′-AGUUACUAUUGAGGAGGCCTT-3′; p300 siRNA#1, 5′-GCACGAACTAGGAAAGAAA-3, p300 siRNA#2, 5′-CGACTTACCAGATGAATTA-3′. All RNAi oligonucleotides were purchased from Shanghai GenePharma Company.

### Western blotting

Cells were lysed with RIPA buffer in the presence of a protease inhibitor cocktail (Roche). Equal amounts of proteins were size fractionated by 6-15% SDS-PAGE. The blots were incubated with specific antibodies against human primary antibodies and the signals were detected using horseradish peroxidase-linked anti-mouse or anti-rabbit conjugates, as appropriate (Zsbio, Beijing, China) and visualized using an ECL detection system (GE Healthcare).

### Co-immunoprecipitation (co-IP)

Cells were lysed in 20 mM Tris-HCl (pH 7.9), 1%NP-40, 150 mM NaCl, 2 mM EDTA, 10% (v/v) glycerol and a protease inhibitor cocktail (Roche). After centrifugation at 12,000*g* for 15 min at 4°C, the cleared lysates were incubated overnight with the respective antibodies or control IgGs and then incubated with protein A/G sepharose beads (GE Healthcare) at 4°C for 2-4 h. The precipitated proteins were eluted with SDS sample buffer and analyzed by SDS-PAGE and western blotting.

### Chromatin immunoprecipitation (ChIP)

HCT116 cells were cross-linked with 1% formaldehyde for 10 min at 37 °C and then washed with cold PBS. The cell pellet was re-suspended in lysis buffer and then sonicated to produce an average DNA length of 500-1,000 bp. The indicated antibodies were added to each sample, and the samples were mixed by rotation at 4°C overnight. Protein A/G Sepharose beads were added to the complexes and incubated for 2 h at 4°C. Protein-DNA complexes were captured on protein A or G Sepharose, washed sequentially with low-salt, high-salt, LiCl, and TE buffer, and then eluted with elution buffer (1% SDS and 0.1 M NaHCO_3_). The cross-links were reversed by heating at 65°C overnight, and the DNA was dissolved in Tris-EDTA buffer and analyzed by qPCR. Precipitated DNA was calculated as the percentage of input DNA. The primer sequences used for ChIP qPCR were as previously described 21.

### *In vitro* methylation assay

Full-length DOT1L plasmids were transfected into HCT116 cells and the DOT1L proteins were immunoprecipitated using Flag-conjugated M2 agarose beads 48 h after transfection. The beads were washed twice with lysis buffer (without NP-40) and then once in methylation buffer (20 mM Tris-HCl pH 8.0, 4mM EDTA, 1 mM PMSF, 0.5 mM DTT). HCT116 nucleosomes and beads were incubated in methylation buffer with or without SAM for 1 h at 30°C before the samples were analyzed by SDS-PAGE and western blotting.

### *In vivo* and *in vitro* acetylation assay

HCT116 cells co-expressing Flag-DOT1L and HA-CBP were lysed in NP-40 buffer [20 mM Tris-HCl (pH 7.9), 150 mM NaCl, 2 mM EDTA, 10% (v/v) glycerol] supplemented with 1% NP-40, protease inhibitor cocktail (Roche), 500 nM TSA, 5 mM sodium butyrate and 10 mM nicotinamide. Flag-DOT1L was then immunopurified from HCT116 cells. The lysates were incubated for 2-4 h at 4°C with M2 anti-Flag beads (Sigma), washed, and subjected to SDS-PAGE and western blotting. *In vitro* acetylation of DOT1L by CBP was monitored using an approach previously used to detect H1K85 acetylation 39.

### *In vivo* and *in vitro* ubiquitination assay

HCT116 cells were co-transfected with the indicated plasmids (Flag-DOT1L, pcDNA or GFP-RNF8) for 48 h. Then, the cells were harvested and lysed in NP-40 buffer. The cell extracts were then incubated with M2 beads for 2 h at 4°C, washed, and subjected to western blotting. *In vitro* ubiquitination of DOT1L by RNF8 was monitored as previously described 40.

### Cell migration and invasion assay

Cell migration assays were performed in 24-well transwell plates with 8-μm polyethylene terephthalate membrane filters (Falcon cell culture insert; Becton-Dickinson) separating the lower and upper culture chambers. HCT116, LoVo or SW480 cells were seeded in the upper chamber at 5×10^4^, 4×10^5^ or 1×10^6^ cells per well in serum-free DMEM, respectively. The bottom chamber contained DMEM with 10% FBS. HCT116 cells were allowed to migrate for 24 h, whereas LoVo and SW480 cells were allowed to migrate for 36 h. After the incubation period, the filter was removed, and non-migrant cells on the upper side of the filter were detached using a cotton swab. Filters were fixed with 4% formaldehyde for 15 min, and the cells located in the lower filter were stained with 0.1% crystal violet for 20 min and counted in three random fields. The quantified results are presented as the means ± SEM. The cell invasion assay was essentially the same as the cell migration assay, except that the membrane filter was coated with Matrigel (BD Biosciences).

### Generation of DOT1L/CBP shRNA-resistant cell lines

Stable DOT1L/CBP knockdown cells were generated by UltraFection 3.0 (4A Biotech), by transfecting shRNA constructs with the DOT1L/CBP siRNA sequences cloned into a pGPU6/Neo vector (GenePharma). The stably transfected cells were selected in neomycin.

### Construction of stable cell lines

HCT116 cells and DOT1L shRNA-resistant cells were transfected with a pHBLV-luci control vector, pHBLV-luci-DOT1L(WT), pHBLV-luci-DOT1L (K358Q) or pHBLV-luci-DOT1L(K358R); CBP shRNA-resistant cells were transfected with a pHBLV-luci control vector. After 36 h, puromycin (Life Technologies) was used to select for stable transfectants; the medium containing puromycin was exchanged every 2-3 days. After 2 weeks, isolated colonies began to appear and by 3 weeks, a pool of puromycin-resistant cells was obtained for further studies.

### *In vivo* metastasis

For tail vein injection-based *in vivo* metastasis assays, cells were injected into the lateral tail vein (1-3×10^6^ cells in 100 μL of phosphate-buffered saline) of 6-week-old male nude mice or NCG mice (Model Animal Research Center, MARC) (n=6 mice per group). For bioluminescence imaging, mice were anesthetized and administered with 150 mg/kg D-luciferin via intraperitoneal injection; 10-15 min after injection, bioluminescence was detected using an IVIS imaging system (PerkinElmer) and analyzed using Living Image software (PerkinElmer). After 4-8 weeks, the mice were sacrificed, and lung tissues were obtained for further histological examination to detect cancer metastasis. All animal experiments were performed under a protocol (IACUC#623918) approved by the Peking University Shenzhen Graduate School Institutional Animal Care (China).

### Tissue samples

Matched primary CRC tissues and their corresponding adjacent normal tissues were collected from colon cancer patients at the First Affiliated Hospital of Shenzhen University and were frozen in liquid nitrogen. These cases were selected based on a clear pathological diagnosis, and the patients had not received preoperative anticancer treatment. Informed consent was obtained from each patient, and the collection of tissue specimens was approved by the Internal Review and Ethics Boards at the First Affiliated Hospital of Shenzhen University.

### Tissue microarray (TMA) and immunohistochemical staining (IHC)

Tissue microarray chips containing 155 pairs of human malignant CRC tissues samples matched to their adjacent normal colon tissue samples and the associated clinicopathological information were purchased from Shanghai Outdo Biotech. Co. Ltd (Shanghai, China). This TMA was used to examine the expression profiles of DOT1L(K358) acetylation or CBP by IHC. For IHC, TMA sections were incubated with an anti-DOT1L(K358) acetylation antibody (1:12000 dilution; PTM BIO Inc) or anti-CBP antibody (1:25 dilution; Santa Cruz) and the resulting staining pattern was scored by two independent pathologists, blinded to the clinical characteristics of the patients. The scoring system was based on the staining intensity and extent, as follows: 0 (negative), 1 (1+), 2 (2+), 3 (3+). Staining positive rate score: 0 (negative), 1 (1-25%), 2 (26-50%), 3 (51-75%), 4 (76-100%). Survival curves were calculated according to the Kaplan-Meier method; survival analysis was performed using the log-rank test. The correlation coefficients among the groups were calculated using the Pearson correlation analysis.

### Quantification and statistical analyses

All data represent the means ± SEM for at least three independent experiments unless otherwise indicated. Statistical significance was determined by unpaired two-tailed Student's *t* tests, and a *p* < 0.05 was considered statistically significant.

## Results

### DOT1L acetylation at K358 positively correlates with CRC stage

**Figure 1:**
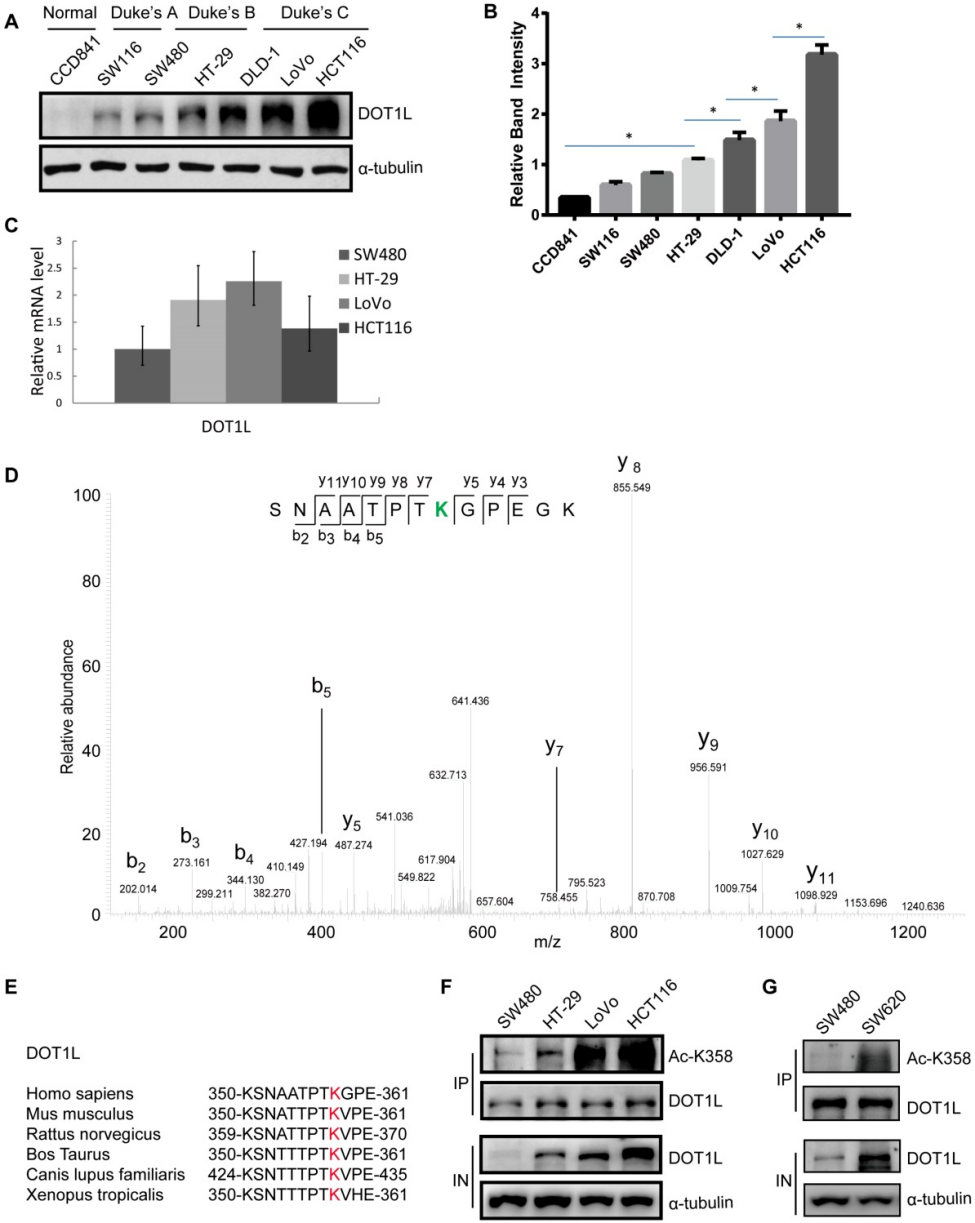
DOT1L acetylation at K358 positively correlates with CRC stage. (A) DOT1L protein levels in normal and colon cancer cell lines at different Duke's stages were examined by western blotting. (B) The quantification of the results of (A) is shown, and the relative DOT1L protein levels are presented as means ± SD (n = 3). *p < 0.05. (C) *DOT1L* mRNA levels in different colon cancer cell lines were extracted with Trizol and were analyzed by real-time PCR. The data are presented as the means ± SD (n = 3). (D) HCT116 cells were transfected with Flag-DOT1L and Flag-immunoprecipitates were separated by SDS-PAGE and stained with CBB. The Flag-DOT1L band was analyzed by mass spectrometry (MS). (E) Alignment of MS-characterized putative DOT1L K358 acetylation residues among different species. (F) DOT1L(K358) acetylation levels in different colon cancer cell lines were analyzed by western blotting. (G) DOT1L(K358) acetylation levels in human primary (SW480) and metastatic (SW620) colon cancer cell lines were analyzed by western blotting.

As DOT1L is a predictor of a poor prognosis in CRC 6, we first aimed to establish the relationship between DOT1L levels and CRC malignancy. We detected DOT1L protein levels in different cell lines originating from normal colon or colon cancer tissues at different stages according to the Duke's staging system and found a strong correlation between DOT1L levels and tumor cell stages (Figure 1A, 1B). Specifically, DOT1L levels were undetectable in normal human CCD841 cells, and increased from the low malignant colon cancer SW480 and SW116 cell lines (Duke's A stage) to the highly malignant colon cancer HCT116, LoVo, DLD-1 and HT-29 cell lines (Duke's B and C stages). Interestingly, DOT1L mRNA levels did not differ according to Duke's stage (Figure 1C). We also detected the DOT1L protein and DOT1L mRNA levels in human colorectal carcinomas and their paired adjacent normal tissues. Here, we found significantly elevated DOT1L protein expression levels in CRC tissues compared with those in paired adjacent normal tissues (Figure S1A). Conversely, *DOT1L* mRNA levels were high in paired adjacent normal tissues (Figure S1B), suggesting that the differential DOT1L protein levels might be due to PTMs.

To identify potential DOT1L PTMs, we performed endogenous DOT1L affinity purification and mass spectrometry (MS) on SW116 and HCT116 cells (Figure S1C). Here, we found that acetylation was the main PTM in both cell lines and the relative abundant of DOT1L acetylation was high in HCT116 cells (Figure S1D). To further explore this PTM, we repeated the MS in Flag-DOT1L-transfected HCT116 cells (Figure S1E); the data suggested that DOT1L might be acetylated at K358 (Figure 1D) and K1228 (Figure S1F). We confirmed K358 acetylation in cells transfected with DOT1L(K358R) or DOT1L(K1228R) plasmids, in which we had mutated the lysine residues to arginine to block acetylation. DOT1L acetylation was notably decreased in these DOT1L(K358R)-transfected cells but unaffected in DOT1L(K1228R)-transfected cells (Figure S1G). Finally, we generated a site-specific antibody against DOT1L(K358) and performed dot blot assays and co-immunoprecipitation (Co-IP) (Figure S1H and Figure S1I) to show that K358 is the main DOT1L acetylation site. This site is located in the DOT1L catalytic domain and is highly conserved from *Xenopus* to humans (Figure 1E).

We then returned to the different CRC cell lines and this time monitored DOT1L acetylation status. Similar to total DOT1L levels, DOT1L(K358) acetylation levels positively correlated with Duke's stage (Figure 1F). In addition, DOT1L and DOT1L(K358) acetylation levels were high in human metastatic CRC cells (SW620) compared with primary CRC cells (SW480) (Figure 1G). These data indicate that DOT1L(K358) acetylation is a critical regulator of DOT1L activity, and acetylation status positively correlates with CRC stage.

### DOT1L acetylation confers DOT1L stability to regulate EMT transcription factor expression

**Figure 2:**
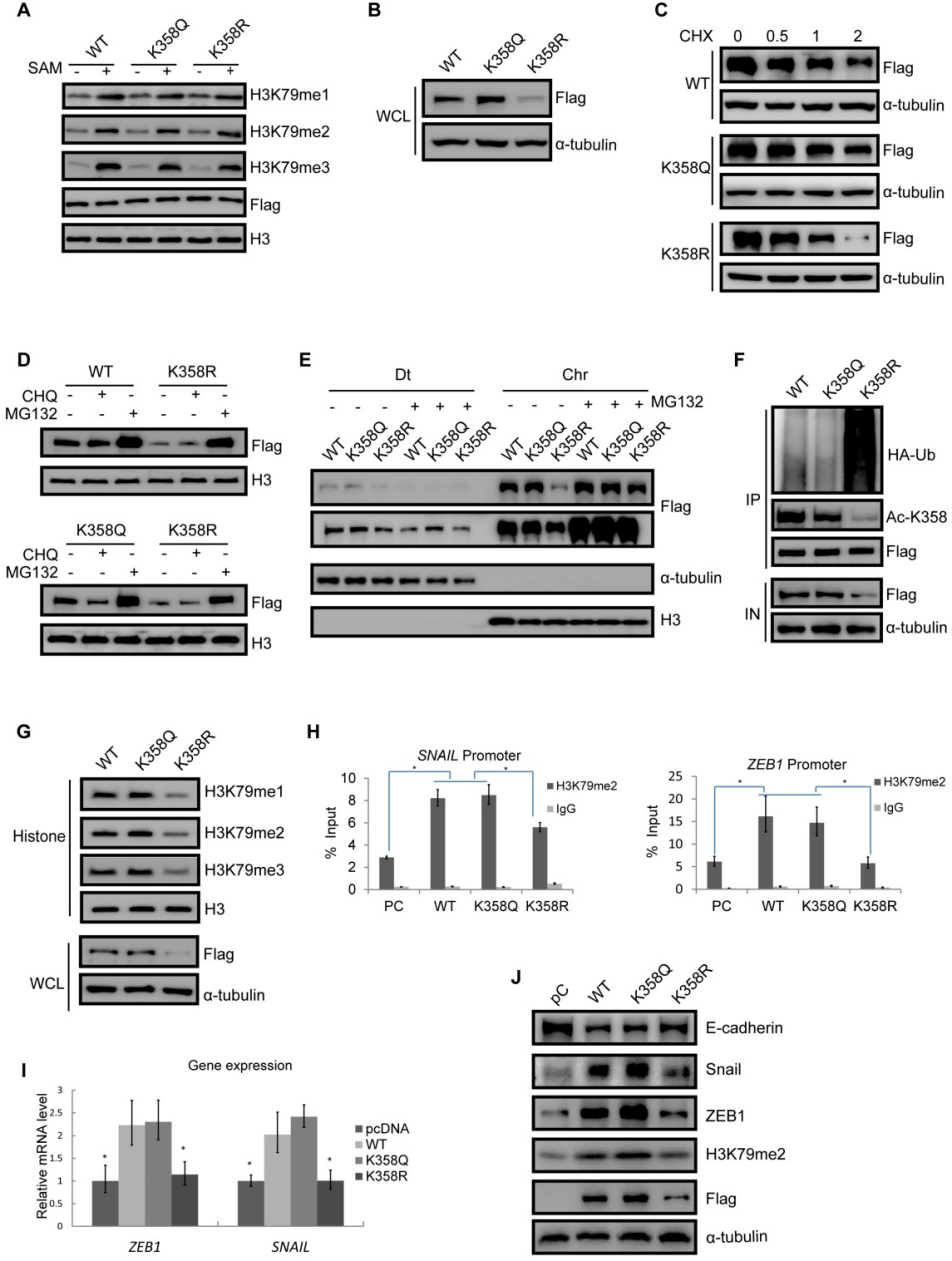
DOT1L acetylation confers DOT1L stability to regulate EMT transcription factor expression. (A) HCT116 cells were transfected with Flag-DOT1L(WT), Flag-DOT1L(K358Q) or Flag-DOT1L(K358R), and the Flag immunoprecipitates were incubated with histones extracted from HCT116 cells separately. Western blotting was performed to detect H3K79me1/2/3 levels. (B) Whole cell lysate (WCL) were extracted from HCT116 cells transfected with Flag-DOT1L(WT), Flag-DOT1L(K358Q) or Flag-DOT1L(K358R), and then analyzed by western blotting with the indicated antibodies. (C) HCT116 cells were transfected with Flag-DOT1L(WT), Flag-DOT1L(K358Q) or Flag-DOT1L(K358R) for 36 h, and then incubated with 20 μg*/*ml cycloheximide (CHX) for the indicated times and analyzed by western blotting. (D) HCT116 cells were transfected with Flag-DOT1L(WT), Flag-DOT1L(K358Q), or Flag-DOT1L(K358R) for 24 h, and subsequently treated with PBS, MG132 (10 μM) or chloroquine (CHQ) (50 μM) for 24 h. WCL were extracted and analyzed by western blotting with the indicated antibodies. (E) HCT116 cells were transfected with Flag-DOT1L(WT), Flag-DOT1L(K358Q) or Flag-DOT1L(K358R) for 24 h, and subsequently treated with PBS or MG132 (10 μM) for 24 h, soluble nucleoplasm proteins (Dt), and chromatin proteins (Chr) were extracted and analyzed by western blotting with the indicated antibodies. (F) DOT1L(K358Q), DOT1L(K358R) or DOT1L(WT) were transfected in HCT116 cells. The lysates were extracted, and DOT1L ubiquitination was detected by co-IP and western blotting with the indicated antibodies. (G) WCL and histones were extracted from HCT116 cells transfected with Flag-DOT1L(WT), Flag-DOT1L(K358Q) or Flag-DOT1L(K358R), and H3K79 methylation was detected by western blotting with the indicated antibodies. (H) ChIP-qPCR showing the level of the indicated proteins recruited to the *SNAIL* (left) and* ZEB1* (right) promoter regions. The data represent the means ± SD (n=3). *p < 0.05. (I)* SNAIL* and *ZEB1* mRNA levels in pcDNA-, DOT1L(WT)-, DOT1L(K358Q)- or DOT1L(K358R)-transfected HCT116 cells were analyzed by RT-qPCR. The data represent the means ± SD (n = 3). *p < 0.05. (J) EMT marker expression was measured by western blotting in HCT116 cells transfected with pcDNA, DOT1L(WT), DOT1L(K358Q) or DOT1L(K358R).

Our data thus far suggest that DOT1L(K358) acetylation is an important regulator of DOT1L activity. To further investigate the effects of DOT1L acetylation, we next aimed to determine whether DOT1L(K358) acetylation status affects DOT1L enzymatic activity and/or stability. By *in vitro* methylation assay, we found that both an acetylation-mimic DOT1L(K358Q) and an acetylation-resistant DOT1L(K358R) mutant retained similar levels of H3K79 methyltransferase activity when incubated with histone 3 (H3) (Figure 2A and Figure S2A). These data suggest that DOT1L acetylation status has no effect on DOT1L methyltransferase activity. Interestingly, DOT1L protein levels were significantly lower in DOT1L(K358R)-transfected cells than in DOT1L(WT) or DOT1L(K358Q)-transfected cells (Figure 2B, Figure S2B), implying that DOT1L(K358) acetylation might ensure DOT1L protein stability.

To confirm our hypothesis, we transfected Flag-tagged DOT1L(K358R), DOT1L(K358Q) or DOT1L(WT) into HCT116 cells in the presence of the protein synthesis inhibitor, cyclohexamide (CHX). Here, DOT1L(K358R) had a much shorter half-life than DOT1L(WT) or DOT1L(K358Q) (Figure 2C). Next, incubation with the proteasome inhibitor MG132, but not the lysosomal inhibitor chloroquinine, spared DOT1L(K358R) from degradation (Figure 2D). Finally, soluble nucleoplasm proteins (Dt), and chromatin proteins (Chr) were detected in DOT1L(WT)-, DOT1L(K358Q)- and DOT1L(K358R)-transfected HCT116 cells incubated with MG132; in this scenario, decreased DOT1L(K358R) binding to chromatin was restored (Figure 2E). These data support that DOT1L acetylation affects DOT1L stability but not enzymatic activity or chromatin binding ability.

We then examined the relationship between DOT1L(K358) acetylation and DOT1L ubiquitination. We first transfected HCT116 cells with Flag-tagged DOT1L(WT), DOT1L(K358Q) or DOT1L(K358R), and then monitored DOT1L acetylation at K358 and ubiquitination by co-IP. Compared with DOT1L(WT) or DOT1L(K358Q), DOT1L acetylation levels decreased while ubiquitination levels increased in DOT1L(K358R)-transfected cells (Figure 2F), indicating that DOT1L deacetylation promotes DOT1L instability by ubiquitination. Consistently, when we detected H3K79 methylation levels according to the differential acetylation status of DOT1L-transfected cells, we found that the DOT1L(K358R) mutation induced a marked decrease in H3K79me1/2/3 levels, whereas the DOT1L(K358Q) and DOT1L(WT) showed sustained H3K79me1/2/3 levels (Figure 2G and Figure S2C).

We next explored the effects of DOT1L stability and accumulation on gene expression. We performed ChIP-qPCR in HCT116 cells transfected with pcDNA, DOT1L(WT), DOT1L(K358Q) or DOT1L(K358R). Here, we found that H3K79me1, H3K79me2, H3K79me3 or DOT1L enrichment in the EMT transcription factor (*SNAIL*, *ZEB1*) promoter regions were decreased in DOT1L(K358R)-transfected cells compared to DOT1L(WT)- or DOT1L(K358Q)-transfected cells (Figure 2H; Figure S2D, E). Consistent with these results, the mRNA levels and protein of these EMT transcription factors were also decreased in DOT1L(K358R)-transfected cells compared to DOT1L(WT)- or DOT1L(K358Q)-transfected cells (Figure 2I, J). The DOT1L(K358R) mutant also abolished the repressed E-cadherin expression evident in DOT1L(WT) or DOT1L(K358Q)-transfected cells, indicating impaired EMT (Figure 2J). In addition, we verified H3K79me2 binding to other reported H3K79 methylation target gene promoter regions (*Pou5F1* and *NANOG*) 6 by ChIP-qPCR in pcDNA-, DOT1L(WT)-, DOT1L(K358Q)- or DOT1L(K358R)-transfected cells. Here, DOT1L enrichment in the *Pou5F1* and *NANOG* promoter regions was decreased in DOT1L(K358R)-transfected cells compared to DOT1L(WT) or DOT1L(K358Q)-transfected cells (Figure S2F). Collectively, these results imply that DOT1L(K358) acetylation protects DOT1L from degradation and promotes H3K79 methylation target genes active transcription, including EMT transcription factor expression. We hypothesize that these events subsequently induce cancer-cell invasion and metastasis.

### DOT1L acetylation regulates CRC migration, invasion and metastasis* in vivo*

**Figure 3:**
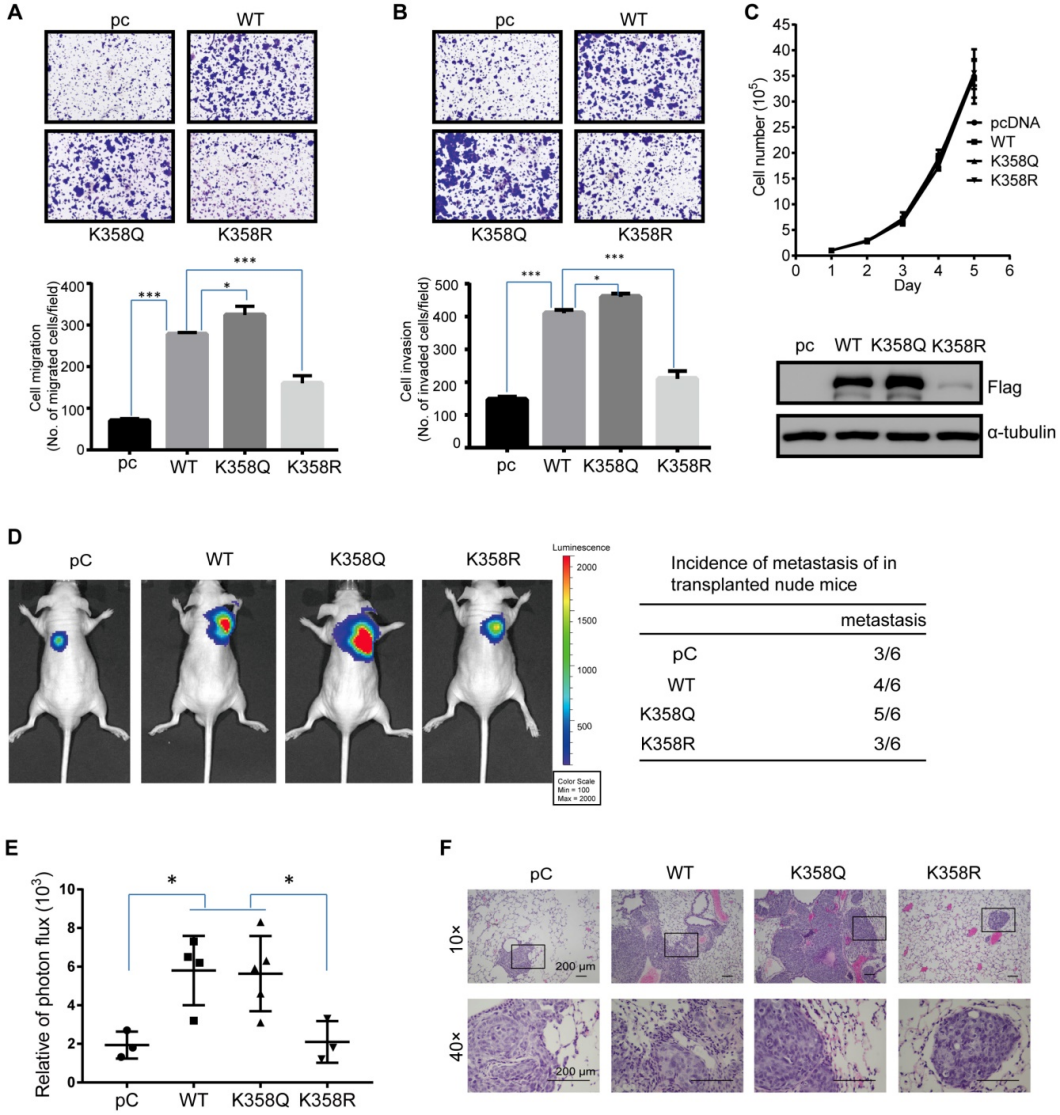
DOT1L acetylation regulates CRC migration, invasion and metastasis* in vivo*. (A, B) Transwell cell migration (A) and matrigel cell invasion (B) assays in HCT116 cells transfected with either pcDNA, DOT1L(WT), DOT1L(K358Q) or DOT1L(K358R) plasmids (upper). The data represent the means ± SEM (n = 3) (lower). *p < 0.05, ***p < 0.001. (C) Comparative growth assay for pcDNA, DOT1L(WT), DOT1L(K358Q) and DOT1L(K358R) overexpressing HCT116 cells. The cell number of each sample was counted at the indicated times. (D, E) HCT116 cells stably expressing pHBLV-luci control (pC), pHBLV-luci-DOT1L(WT) pHBLV-luci-DOT1L(K358Q) or pHBLV-luci-DOT1L(K358R) plasmids were injected intravenously *via* the tail vein into 6-week-old male nude mice (n = 6 mice per group). Lung metastasis was monitored by bioluminescent imaging after 7 weeks of injection. Representative *in vivo* bioluminescent images and the incidence of lung metastasis from the different groups are shown (D); the bioluminescent quantitation of lung metastases is given (E). *p < 0.05 compared with the DOT1L(WT) and DOT1L(K358Q). The data represent the means ± SD. (F) Representative lung metastasis specimens were sectioned and stained with H&E. Scale bars: 200 μm.

To demonstrate the functional significance of DOT1L(K358) in CRC progression, we next assessed whether DOT1L(K358) acetylation contributes to CRC migration, invasion and metastasis. Firstly, we determined the role of DOT1L on CRC cell migration and invasion (Figure S3A, B). Here, we observed decreased cell migration and invasion ability in DOT1L-knockdown HCT116 cells, suggesting the important role of DOT1L in CRC cell migration and invasion. Next, we established a transwell cell migration and a matrigel-coated invasion assay in which we over-expressed pcDNA, DOT1L(WT), DOT1L(K358Q) or DOT1L(K358R) in HCT116 cells. We detected a significantly lower degree of cellular migration and invasion by pcDNA and DOT1L(K358R) cells than DOT1L(WT) and DOT1L(K358Q) cells (Figure 3A, B). Conversely, DOT1L acetylation did not significantly affect HCT116 cell growth (Figure 3C), indicating that the observed changes in migration and invasion activities did not result from the effects of DOT1L acetylation on cell growth. We confirmed decreased cell migration and invasion in the context of pcDNA and DOT1L(K358R) compared to DOT1L(WT) and DOT1L(K358Q) in LoVo and SW480 cells (Figure S3C-F).

Having shown that down-regulated DOT1L(K358) acetylation is associated with a decrease in cancer-cell migration and invasion *in vitro* (Figure 3A, B; Figure S3C-F), we determined the effects of DOT1L(K358R) on CRC metastasis *in vivo* using a bioluminescence imaging approach. We stably transfected HCT116 cells with pHBLV-luci control (pC), pHBLV-luci-DOT1L(WT), pHBLV-luci-DOT1L(K358Q) or pHBLV-luci-DOT1L(K358R), and then injected these cells into male nude mice (n = 6 mice per group) via the tail vein. We then monitored seeding lung metastasis by quantitative bioluminescence imaging, 7 weeks after cell injection. Compared with the empty plasmid, pHBLV-luci-DOT1L(WT) and pHBLV-luci-DOT1L(K358Q) produced a high level of metastatic HCT116 tumors in the lungs whereas pHBLV-luci-DOT1L(K358R) produced a relatively low level of lung metastatic tumors (Figure 3D, E). We verified the presence of lung metastases by histological staining and confirmed an increased level of lung metastases in the DOT1L(WT) and DOT1L(K358Q) compared to control and DOT1L(K358R) conditions (Figure 3F). Conversely, DOT1L downregulation by DOT1L shRNA decreased the extent of lung metastasis (Figure S3G-I); this reduction in cell metastasis could be restored upon over-expressing DOT1L(WT) and DOT1L(K358Q), but not DOT1L(K358R) (Figure S3G-I). Collectively, these experiments indicate that DOT1L acetylation correlates with cancer-cell migration, invasion and metastasis *in vivo*.

### CBP mediates DOT1L acetylation *in vivo* and *in vitro* and confers DOT1L stability

**Figure 4:**
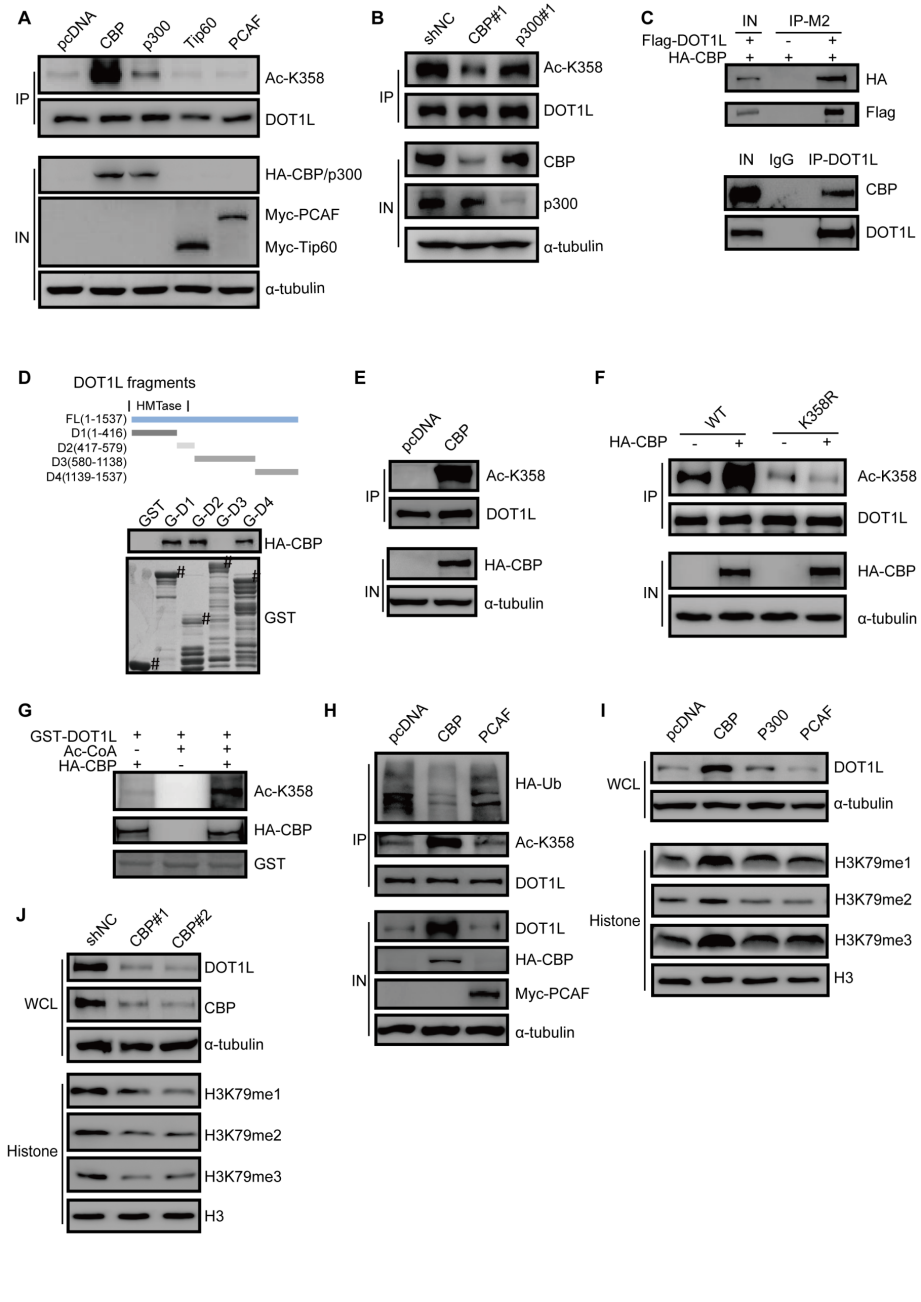
CBP mediates DOT1L acetylation *in vivo* and *in vitro* and confers DOT1L stability. (A) DOT1L(K358) acetyltransferase screen by over-expressing a series of acetyltransferases in HCT116 cells followed by western blotting. (B) HCT116 cells were transfected with CBP or p300 siRNAs prior to detecting DOT1L acetylation levels by immunoprecipitation (IP). (C) Endogenous and an exogenous co-IP to detect the interaction between DOT1L and CBP in HCT116 cells. (D) Purified GST or GST-DOT1L fragments were incubated with HA-CBP prior to western blotting to detect the HA-CBP and GST-DOT1L fragment interactions. (E) *In vivo* acetylation assay. HCT116 cells were transfected with pcDNA or HA-CBP, and the DOT1L immunoprecipitates were analyzed by western blotting with the indicated antibodies. (F) HCT116 cells were co-transfected with pcDNA or HA-CBP and DOT1L(WT) or DOT1L(K358R), then DOT1L acetylation levels were detected by western blotting. (G) HCT116 cells were transfected with control pcDNA or HA-CBP, and the HA-CBP immunoprecipitates were incubated with GST-DOT1L. DOT1L acetylation levels were analyzed by western blotting with the indicated antibodies. (H) HCT116 cells co-transfected with control pcDNA, HA-CBP or Myc-PCAF, and DOT1L immunoprecipitates were analyzed by western blotting with the indicated antibodies. (I) Whole cell lysate (WCL) and histones were extracted from HCT116 cells transfected with pcDNA, HA-CBP, HA-p300 or Myc-PCAF, and then analyzed by western blotting with the indicated antibodies. (J) Whole cell lysate (WCL) and histones were extracted from HCT116 cells transfected with non-specific siRNA negative control shRNA (shNC), CBP#1 or CBP#2 siRNAs and then analyzed by western blotting with the indicated antibodies.

Having established DOT1L(K358) acetylation as a critical controller of DOT1L associated with CRC invasion and metastasis, we aimed to identify the specific acetyltransferase by screening a series of acetyltransferases (including CBP, p300, PCAF, and Tip60). Here, we found that CBP dramatically increased DOT1L acetylation levels, while p300 had only a mild effect (Figure 4A). By contrast, the other acetyltransferases tested could not catalyze DOT1L acetylation (Figure 4A). Similar results were produced when using LoVo cells (Figure S4A).

In addition, DOT1L acetylation levels significantly decreased upon CBP, but not p300, knockdown, suggesting that CBP is the predominant DOT1L(K358) acetyltransferase in HCT116 cells (Figure 4B). Exogenous and endogenous co-IP assays in HCT116 cells confirmed that DOT1L interacted with CBP (Figure 4C, Figure S4B), and GST pulldown assay supported a direct interaction between the two proteins (Figure 4D). Furthermore, *in vivo* and *in vitro* acetylation assays showed that CBP could indeed catalyze DOT1L(K358) acetylation (Figure 4E-G; Figure S4C).

We next investigated whether CBP-mediated DOT1L acetylation positively regulates DOT1L stability. We observed decreased DOT1L ubiquitination and increased DOT1L and catalyzed H3K79me1/2/3 in HCT116 cells transfected with HA-CBP compared to pcDNA (Figure 4H, I). Finally, CBP knockdown by siRNA resulted in decreased DOT1L and H3K79 methylation levels (Figure 4J). Next, we performed migration and invasion assays and *in vivo* metastasis assays in CBP-knockdown cells. Consistent with our observations in DOT1L(K358R)-transfected cells (Figure 3A-F), cellular migration and invasion levels were decreased in CBP-knockdown cells compared to control cells (Figure S4D, E). We also detected a reduced level of lung metastases following injection of CBP-knockdown cells compared to control cells (Figure S4F, G). Collectively, these data suggest that CBP catalyzes DOT1L acetylation and upregulates its stability. Moreover, CBP functions as a DOT1L acetyltransferase and is essential for CRC migration, invasion and metastasis.

### E3 ligase RNF8 ubiquitinates DOT1L prior to degradation

**Figure 5:**
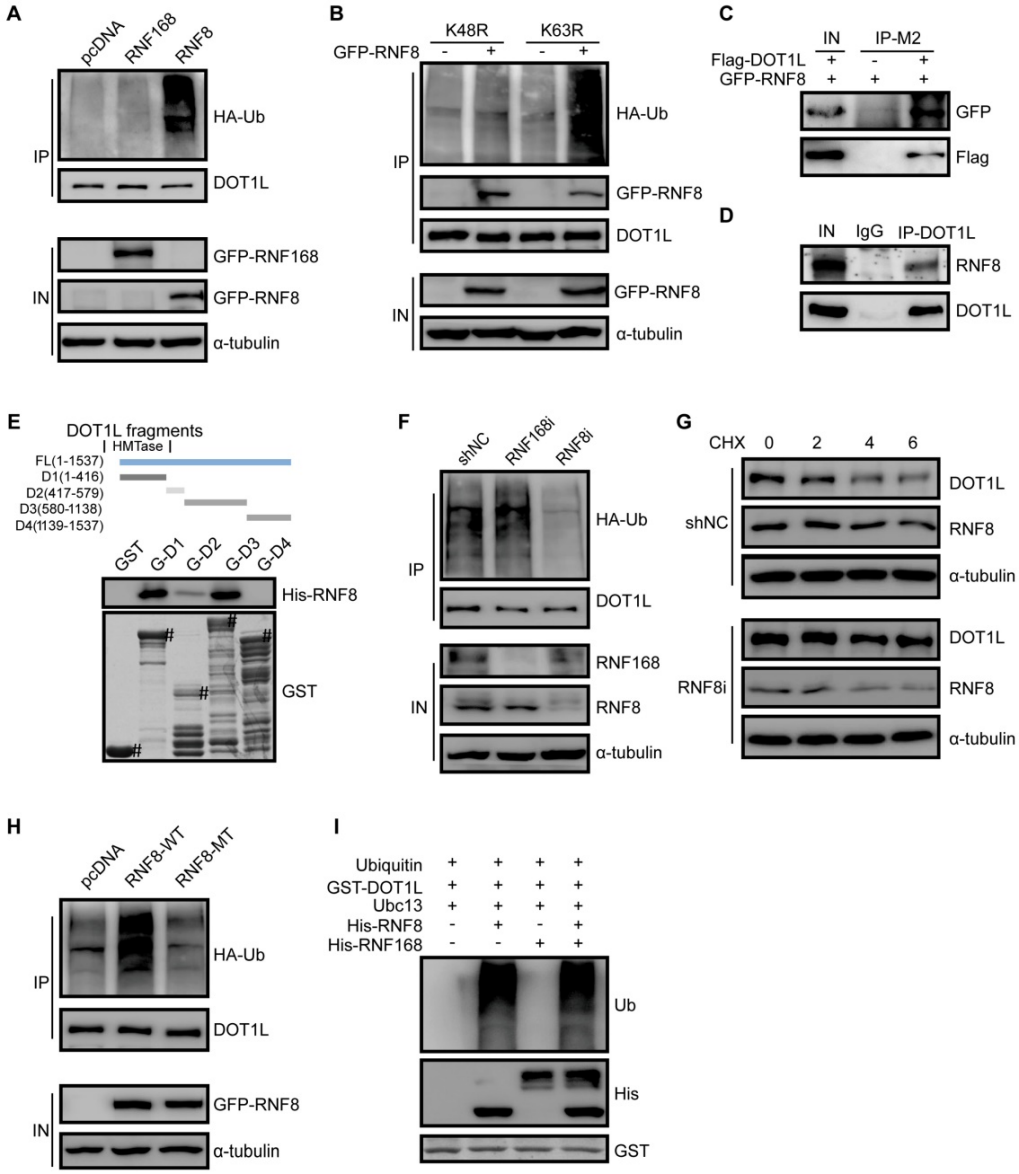
E3 ligase RNF8 ubiquitinates DOT1L prior to degradation. (A) HCT116 cells were transfected with HA-tagged ubiquitin (Ub) and control pcDNA, GFP-RNF8 or GFP-RNF168 separately, and the cell lysates were subjected to western blotting with the indicated antibodies to detect DOT1L ubiquitination. (B) HCT116 cells were transfected with control pcDNA or GFP-RNF8 together with HA-Ub(K48R) or HA-Ub(K63R), and western blotting was performed with the indicated antibodies to detect DOT1L ubiquitination. (C, D) Nuclear proteins were extracted from HCT116 cells, and endogenous (C) and exogenous (D) co-IPs were performed to detect the interaction between DOT1L and RNF8. (E) Purified GST or GST-DOT1L fragments were incubated with His-RNF8. Western blotting was performed to detect His-RNF8 protein levels, and CBB staining was performed to detect GST or GST-tagged proteins (#). (F) HCT116 cells were transfected with a non-specific shRNA negative control (shNC), RNF8 siRNA or RNF168 siRNA for 72 h. DOT1L immunoprecipitates were subjected to western blotting with the indicated antibodies to detect DOT1L ubiquitination. (G) HCT116 cells were transfected with shNC or RNF8 siRNA. The protein lysates were extracted from 0-6 h following CHX treatment and the DOT1L, RNF8 and α-tubulin protein levels were examined by western blotting. (H) HCT116 cells were co-transfected with HA-Ub, pcDNA, GFP-RNF8(WT) or enzymatically defective GFP-RNF8 (RNF8-MT), and the DOT1L immunoprecipitates were subjected to western blotting with the indicated antibodies. (I) Combinations of GST-DOT1L, UBE1 (E1), Ubc13 (E2), His-RNF8 (E3), and His-RNF168 (E3) were incubated at 37°C for 1 h, and detected by western blotting.

We next screened the possible E3 ligases responsible for DOT1L degradation, and found that DNA damage repair related E3 ligases RNF8 might be involved in this process. To confirmed it, we transfected GFP-RNF8 or GFP-RNF168 plasmids into HCT116 and LoVo cells and detected the changes in DOT1L ubiquitination by co-IP. Indeed, over-expression of RNF8, but not RNF168, increased DOT1L ubiquitination levels (Figure 5A, Figure S5A). Specifically, RNF8 over-expression increased K48-linked but not K63-linked DOT1L ubiquitination (Figure 5B). Furthermore, exogenous and endogenous co-IP assays in HCT116 cells and LoVo cells showed that DOT1L could co-precipitate with RNF8 (Figure 5C, D and Figure S5B). We confirmed that this interaction was direct by GST pull-down assay: fragments corresponding to the DOT1L 1-416aa, 417-579aa and 580-1138aa fragments interacted with RNF8, except for DOT1L C-terminal fragment (1139-1537aa) (Figure 5E). Finally, down-regulating RNF8, but not RNF168, by siRNA markedly reduced DOT1L ubiquitination levels (Figure 5F) and thus increased the DOT1L half-life compared to siRNA controls (Figure 5G).

To further study that RNF8 acts as a DOT1L E3 ligase, we compared DOT1L ubiquitination levels in HCT116 cells transfected with control (pcDNA), RNF8(WT) or RNF8(MT) plasmids, whereby RNF8(MT) is enzymatically defective. Compared with pcDNA and RNF8(MT), only RNF8(WT) could increase DOT1L ubiquitination levels (Figure 5H). Additionally, *in vivo* ubiquitination assay in HT-29 and LoVo cells showed that RNF8 increased DOT1L ubiquitination compared with pcDNA (Figure S5C, D). Further *in vitro* ubiquitination assays confirmed that RNF8 is a bona fide DOT1L E3 ligase (Figure 5I, Figure S5E).

### DOT1L acetylation protects DOT1L from degradation *via* preventing the interaction between RNF8 and DOT1L

**Figure 6:**
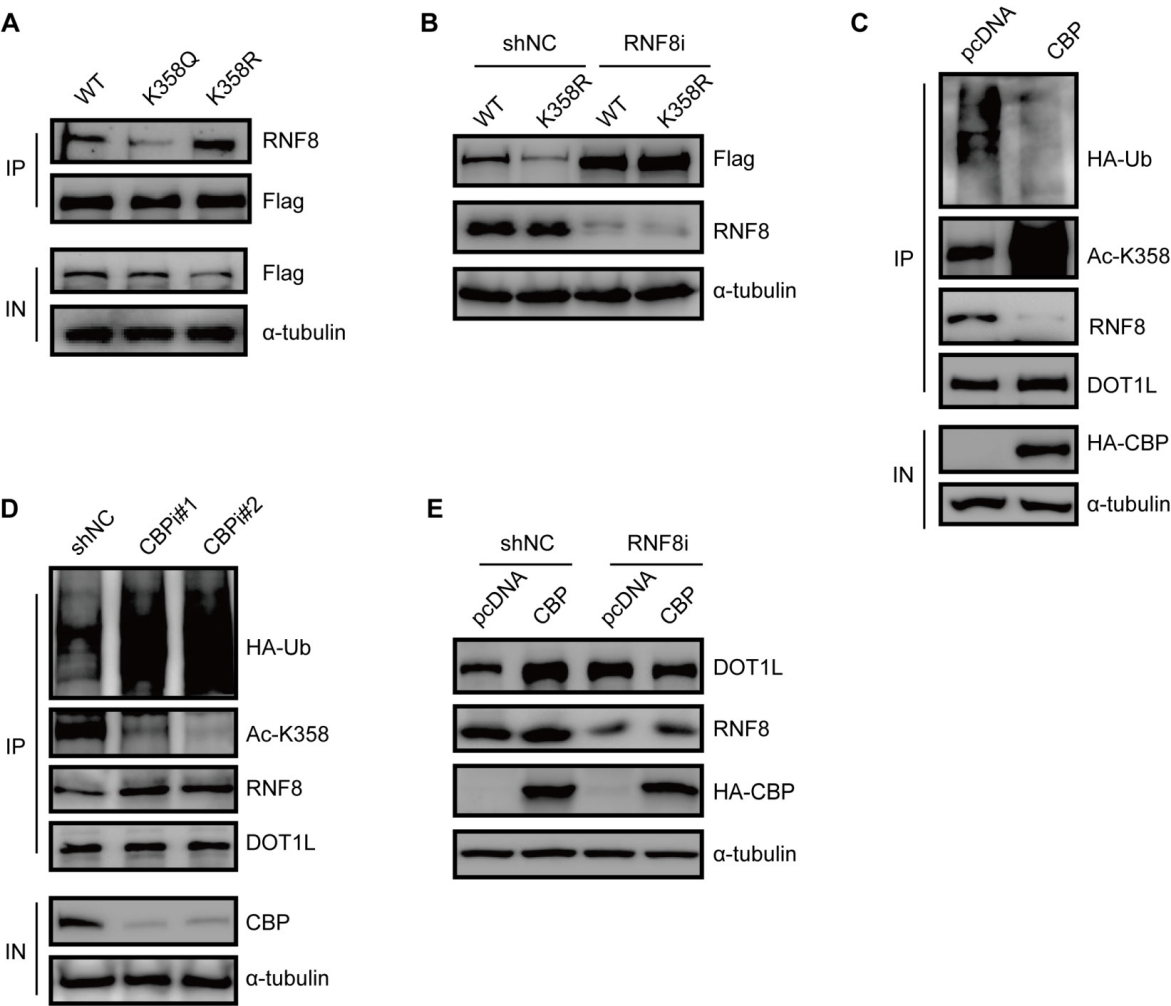
** DOT1L acetylation protects DOT1L from degradation *via* preventing the interaction between RNF8 and DOT1L. (A)** Co-IPs were performed to detect the interaction between RNF8 and DOT1L(WT), DOT1L(K358Q) or DOT1L(K358R) in HCT116 cells. **(B)** HCT116 cells were transfected with a non-specific shRNA negative control (shNC) or an RNF8 siRNA for 24 h followed by transfection with Flag-DOT1L(WT) and Flag-DOT1L(K358R) for 48 h. The whole cell lysates were analyzed by western blotting with the indicated antibodies. **(C)** HCT116 cells were transfected with pcDNA or HA-CBP, and then subjected to immunoprecipitation (IP) and western blotting with the indicated antibodies. **(D)** HCT116 cells were transfected with non-specific shNC or CBP siRNAs, and the proteins were analyzed by western blotting with the indicated antibodies. **(E)** HCT116 cells were transfected with shNC or RNF8 siRNA for 24 h, and then transfected with HA-CBP for 48 h. The whole cell lysates were extracted and analyzed by western blotting with the indicated antibodies.

To gain a mechanistic insight into DOT1L acetylation-dependent stability, we monitored the RNF8-DOT1L interaction according to DOT1L acetylation status. Compared with DOT1L(WT), the interaction between DOT1L and RNF8 was impaired upon transfection with the DOT1L(K358Q) mutant, but enhanced upon transfection with the DOT1L(K358R) mutant (Figure 6A). Upon RNF8 knockdown by siRNA, DOT1L(K358R) showed enhanced stability (Figure 6B). We also found that the DOT1L-RNF8 interaction decreased upon HA-CBP over-expression in HCT116 cells compared to control cells (Figure 6C). Correspondingly, the RNF8-DOT1L interaction and DOT1L ubiquitination levels were markedly increased in CBP-knockdown HCT116 cells (Figure 6D). GST pulldown assay detected no direct interaction between RNF8 and CBP (Figure S6A). Finally, we treated HCT116 cells with RNF8 or control siRNA and then transfected the cells with pcDNA or HA-CBP. The increased of DOT1L levels elicited by CBP were blocked in RNF8-knockdown cells (Figure 6E). Together, these results indicate that CBP-mediated DOT1L(K358) acetylation affects the RNF8-DOT1L interaction to protect DOT1L from proteasomal-dependent degradation.

### DOT1L acetylation levels positively correlates with CBP expression and is associated with CRC metastasis and progression

**Figure 7:**
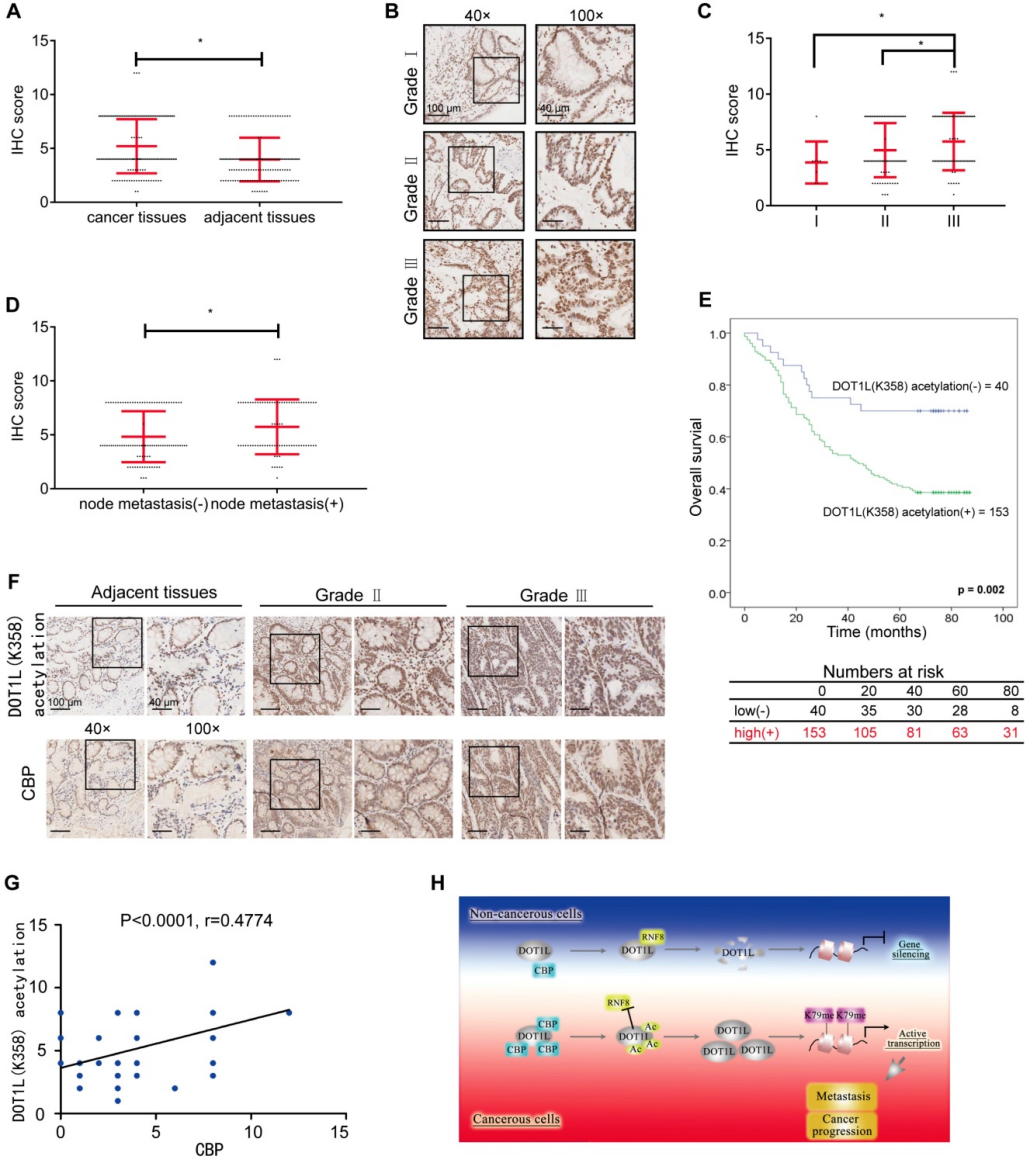
DOT1L acetylation levels positively correlates with CBP expression and is associated with CRC metastasis and progression. (A) DOT1L(K358) acetylation staining on tissue microarrays (TMAs) containing 155 normal and CRC tissues. The relative DOT1L(K358) acetylation levels were compared between normal and CRC tissues (Student's *t*-test, n =155; the data represent the means ± SD, error bars are shown in red; *P<0.05). (B) IHC staining of TMAs containing 193 colon carcinoma samples (grades I, II, III) for DOT1L(K358) acetylation levels. Representative images of anti-DOT1L(K358) acetylation staining. Scale bars: 100 μm (left); 40 μm (right). (C) DOT1L(K358) acetylation staining scores were determined by evaluating the extent and intensity of immune-positivity and were analyzed by two-tailed unpaired student's *t*-test; the data represent the means ± SD, error bars are shown in red; *p < 0.05. (D) The relative DOT1L(K358) acetylation levels were compared between colon adenocarcinoma tissues with and without lymph node metastasis. The data were analyzed by Student's *t*-test, n =193; the data represent the means ± SD, error bars are shown in red; *P<0.05. (E) Kaplan-Meier curve showing the percentage overall survival of patients with colon cancer, stratified by DOT1L(K358) acetylation expression. (F) Samples from adjacent normal tissues and CRC cancers were immuno-stained with antibodies against DOT1L(K358) acetylation or CBP. Representative images are shown. Scale bars: 100 μm (left); 40 μm (right). (G) The CBP scores were plotted against the DOT1L(K358) acetylation scores, and the correlation coefficients were calculated by Pearson correlation analysis. (H) A proposed model of the DOT1L(K358) regulatory pathway in CRC carcinogenesis. Excessive CBP-mediated DOT1L acetylation in cancerous cells protects DOT1L from degradation *via* RNF8 and induces DOT1L enrichment. This effect enables H3K79 methylation of target genes and transcriptional activation of EMT transcription factors. The overall effect is promotion of tumorigenesis and metastasis.

Having dissected the relevance of DOT1L(K358) acetylation on tumorigenesis and metastasis in cells and mice, we wanted to know whether there exists any correlation between DOT1L(K358) acetylation levels and tumor incidence or metastasis in humans. To this aim, we examined DOT1L(K358) acetylation levels in human CRC tissue samples by IHC. First, we analyzed DOT1L(K358) acetylation levels in 155 pairs of human malignant CRC tissues and matched adjacent noncancerous colon tissues. IHC staining confirmed that DOT1L(K358) acetylation levels in CRC tissues were significantly higher than in the corresponding adjacent normal tissues (Figure 7A; Table S1).

Next, we examined how DOT1L(K358) acetylation levels vary by CRC grade by IHC staining of a human tissue array including 193 CRC samples from patients with grade I, II, or III CRC. Remarkably, we found that DOT1L(K358) acetylation levels positively correlated with the histological tumor grade, suggesting that DOT1L(K358) acetylation levels progressively increase during CRC progression (Figure 7B, C; Figure S7A; Table S2-3). Interestingly, DOT1L(K358) acetylation levels in primary colon adenocarcinomas with metastasis were significantly higher than in those without metastasis (Figure 7D). Finally, we observed reduced overall survival in patients with high DOT1L(K358) acetylation levels (Figure 7E), indicating that DOT1L(K358) acetylation status might be an informative prognostic indicator.

Considering that DOT1L(K358) acetylation is mediated by CBP in cells, we finally explored whether these factors also correlate with CRC incidence or metastasis in human patients. We first examined CBP protein and DOT1L(K358) acetylation levels in the human tissue arrays and found that the CBP expression in CRC tissues was significantly higher than in the corresponding cancer-adjacent normal tissues and positive correlates with CRC progression (Figure S7B, C; Table S4-6). In addition, CBP expression in primary colon adenocarcinomas with metastasis was significantly higher than in those without metastasis (Figure S7D). Finally, we found a strong, positive correlation between CBP and DOT1L(K358) acetylation levels in CRC (Figure 7F, G). Taken together, these results indicate that DOT1L(K358) acetylation levels are mediated by CBP, and have a central role in facilitating CRC development, progression and metastasis (Figure 7H).

## Discussion

The present study has identified that CBP-mediated DOT1L acetylation at K358 is a critical PTM that maintains DOT1L stability. This stability ensures H3K79 methylation, EMT transcription factor expression and subsequent CRC migration and invasion. Mutating the DOT1L K358 site to R358 renders DOT1L susceptible to degradation *via* an interaction with the E3 ligase RNF8 and this process inhibits cancer-cell metastasis. Importantly, we found that high DOT1L(K358) acetylation levels in patients correlate with CRC metastasis and progression, which also positively correlate with high CBP expression. These data indicate the relevance of DOT1L(K358) acetylation in human CRC progression and prognosis.

Many studies have suggested that DOT1L-regulated H3K79 methylation mainly depends on DOT1L recruitment to specific histone modification regions. For example, histone 2B ubiquitination (H2Bub) can interact directly or indirectly with DOT1L to alter chromatin structure and facilitate DOT1L-mediated H3K79 methylation 23, 41. One report showed that DOT1L binds the ubiquitinated nucleosome and that DOT1L activity is stimulated by H2B-Ub 42, 43. In addition, several DOT1L-containing complexes have been described that might control the location and extent of H3K79 methylation across the human genome 44. AF9 acts as a bridge protein to recruit DOT1L to transcriptionally active genes decorated with H3K9ac and helps maintain the open chromatin state 24. AF10 recognizes unmodified H3K27, but not methylated H3K27, and recruits DOT1L to specific genomic regions 25. Despite these advances, how DOT1L stability is regulated and how its stability affects H3K79 methylation, had not been previously reported. We report for the first time that DOT1L stability is regulated independently of the crosstalk between histone modification and protein recruitment, namely, by acetylation and proteasomal-mediated degradation. Further work that focuses on DOT1L deacetylation to destabilize DOT1L, which might be a premiered negatively regulatory mechanism, will expand our understanding on the DOT1L regulatory mechanisms.

Our key finding is that DOT1L stability is regulated by acetylation: when DOT1L is acetylated at K358, a protein interaction with RNF8 is lost and DOT1L(K358) protein levels are stabilized. Acetylation is a major PTM and is widely associated with modulating protein stability, as acetylation can prevent protein degradation by inhibiting ubiquitination. Such acetylation-induced changes in degradation could be due to direct competition for the same lysine residues to be acetylated or ubiquitylated 45 Alternatively, acetylation might prevent protein degradation *via* altering the overall protein structure and thus affect protein-protein interactions 46. Here, we found that DOT1L acetylation stabilizes DOT1L by impairing the interaction between DOT1L and RNF8. This protection from RNF8-induced degradation might be as a result of a DOT1L conformational change that prevents RNF8 binding. Indeed, our GST-pulldown data showed that RNF8 directly interacts with the DOT1L N-terminus (1-416aa) (Figure 5E), which encompasses the DOT1L acetylation site. Further work remains to determine whether protection from RNF8-induced degradation may be as a result of a DOT1L conformational change upon acetylation.

RNF8/RNF168 is one of very few E3 ligases that can mediate both the K48 and K63 forms of ubiquitination 47-49. Early studies showed that RNF8 is involved in K63-linked ubiquitination and protein recruitment to double strand breaks 40, 50-52. One study found that RNF8 can promote EMT activators in breast cancer cells by regulating the K63-linked ubiquitination of Twist, which in turns regulates Twist nuclear localization and protein stability 53, 54. Other reports have demonstrated that RNF8 can regulate degradation via K48-linked polyubiquitination of protein targets, including FOXM1, 53BP1 and JMJD2A 55-57. Consistently, we found that RNF8 promotes DOT1L K48-linked poly-ubiquitination and targets it for degradation (Figure 5B). Together, these findings depict the complex biology of RNF8 and suggest that the effects of RNF8 on cancer migration and invasion likely depend on the RNF8 target protein.

We identified DOT1L as a new CBP substrate, providing further support for CBP-mediated acetylation in cancer-cell invasion and metastasis 58, 59. CBP is a transcriptional activator and acetyltransferase; it is a large multi-domain protein that can interact with >400 different proteins 60. CBP-mediated acetylation of non-histone substrates has been widely implicated in cancer development 60, 61. Because of the high sequence homology observed between CBP and p300, the two proteins are collectively referred to as p300/CBP 62, 63. Interestingly, we found that CBP alone is the predominant DOT1L acetyltransferase. This specificity might be explained by the different structural organization and binding partners between CBP and p300 64-66. Further work regarding the structural conformation of CBP-DOT1L binding is now needed to confirm the specificity of CBP catalytic activity towards DOT1L. Together, the present study provides yet another example of how protein regulation by CBP contributes to CRC metastasis.

ChIP-chip analyses of yeast, mouse, fly, and human genomes revealed that H3K79 methylation marks are localized within the region of transcribed genes 12, 67, 68. *BCAT1* is one DOT1L target gene regulated by H3K79 methylation and is responsible for DOT1L-mediated cell migration in breast cancer cells 69. The DOT1L-c-Myc complex functions as a transcriptional activator to increase the expression of EMT regulators. Up-regulated H3K79 methylation on the *SNAIL*, *ZEB1* and *ZEB2* promoters enhances EMT-induced breast-cancer invasion and metastasis 21. Here, we found that DOT1L(K358) acetylation results in upregulated H3K79 methylation and EMT transcription factor expression and subsequent CRC migration and invasion (Figure 2G-J, Figure 3A, B). This finding provides further support that DOT1L regulates cell migration and invasion *via* target gene H3K79 methylation.

To determine the relevance of our molecular findings in the context of human CRC, we assessed how DOT1L(K358) acetylation levels correlate with CBP in CRC. We found that DOT1L(K358) acetylation promotes CRC progression and metastasis and correlates with a poor patient prognosis. Furthermore, we found a significant correlation between DOT1L(K358) acetylation levels and CBP. These observations are consistent with a role for CBP in maintaining DOT1L acetylation in colon cancer cells. High CBP expression is a known predictor of poor survival in CRC 70. Consistently, we also found that CBP expression correlates with tumor stage and poor prognosis in CRC, further strengthening our data that CBP-mediated DOT1L acetylation at K358 has a vital role in tumor progression and prognosis. The therapeutic potential of CBP specific inhibitors has been reported 71, 72; here, our finding further highlights the prospect of CBP inhibitors in cancer therapy, especially in cancers highly expressing DOT1L/CBP. Moreover, CBP inhibitors and DOT1L inhibitors (that inhibit DOT1L enzymatic activity) or other targeted chemotherapies provide new opportunities for combinatorial CRC treatment and potentially other cancers such as leukemia.

In conclusion, our study has identified a new regulatory network involving DOT1L whereby RNF8-mediated DOT1L instability is controlled by CBP to modulate H3K79 methylation levels and EMT transcription factor expression. This scenario might open new avenues to design effective therapeutics to combat hyper-expressed DOT1L-driven tumors, such as CRC. Overall, we consider that CBP/DOT1L could serve as a prognostic biomarker for CRC and help guide tumor combination therapy targeting epigenetic modulators.

## Supplementary Material

Supplementary figures and tables.

## Abbreviations

CRC: colorectal cancer
EMT: epithelial-mesenchymal transition
ChIP: chromatin immunoprecipitation analysis
ZEB1: zinc finger E-box binding homeobox 1
CBP: CREB-binding protein
RNF8: RING finger 8
IHC: immunohistochemical
