# Supplementary material for: CBP mediated DOT1L acetylation confers DOT1L stability and promotes cancer metastasis
Source: Theranostics. 2020 Jan 1;10(4):1758–76. doi: 10.7150/thno.39013 (PMC6993218; doi:10.7150/thno.39013)
Supplement: Supplementary file 1 — Supplementary figures and tables. [file thnov10p1758s1.pdf]

Figure S1 DOT1L acetylation at K358 positively correlates with CRC stage. Related to Figure 1.

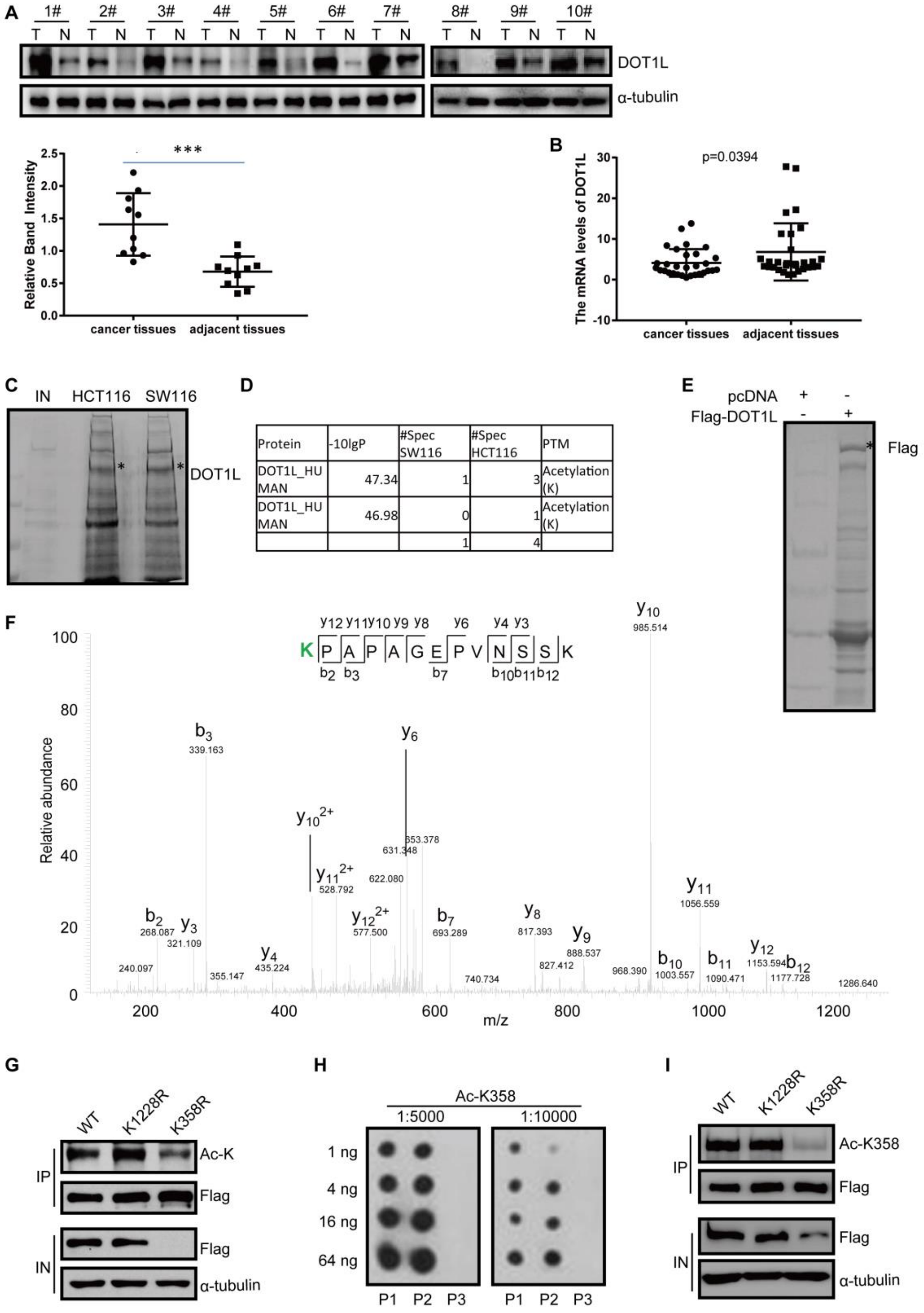

(A) Analysis of DOT1L protein levels in human colorectal carcinomas and their paired adjacent normal tissues (n = 10) (upper). Quantified results are presented as the means ± SD (n = 3). ***$p < 0.001$ (lower). (B) *DOT1L* mRNA levels in 30 human colorectal carcinomas and their paired adjacent normal tissues were analyzed by RT-qPCR. The data are presented as the means ± SD (n = 30). (C, D) DOT1L-immunoprecipitates from HCT116 and SW116 cells were separated by SDS-PAGE and stained with CBB (C). The DOT1L bands (*) in (C) were analyzed for acetylation by mass spectrometry (D). (E, F) Co-immunoprecipitation products extracted in pcDNA- or Flag-DOT1L-transfected HCT116 cells after adding anti-Flag antibody were separated by SDS–PAGE and stained with CBB (E). The Flag-DOT1L bands (*) in (E) were analyzed for acetylation by mass spectrometry (F). (G) DOT1L(K1228R) or DOT1L(K358R) acetylation-abrogated mutants were transfected into HCT116 cells and detected with anti-acetyl-lysine antibodies. (H) Dot blot analysis of the DOT1L(K358) acetylation antibody. P1 refers to acetyl-DOT1L(K358) peptide 1 (NAATPT-(acetyl)K-GPEGKC); P2 refers to acetyl-DOT1L(K358) peptide 2 (CATPT-(acetyl)K-GPEGKVA); P3 refers to unmodified-DOT1L(K358) peptide 3 (CNAATPTKGPEGKVA). (I) HCT116 cells were transfected with Flag-DOT1L mutant plasmids DOT1L(K358R) or DOT1L(K1228R) and then analyzed using an anti-DOT1L(K358) acetylation antibody.

Figure S2 DOT1L acetylation confers DOT1L stability to regulate EMT transcription factor expression. Related to Figure 2.

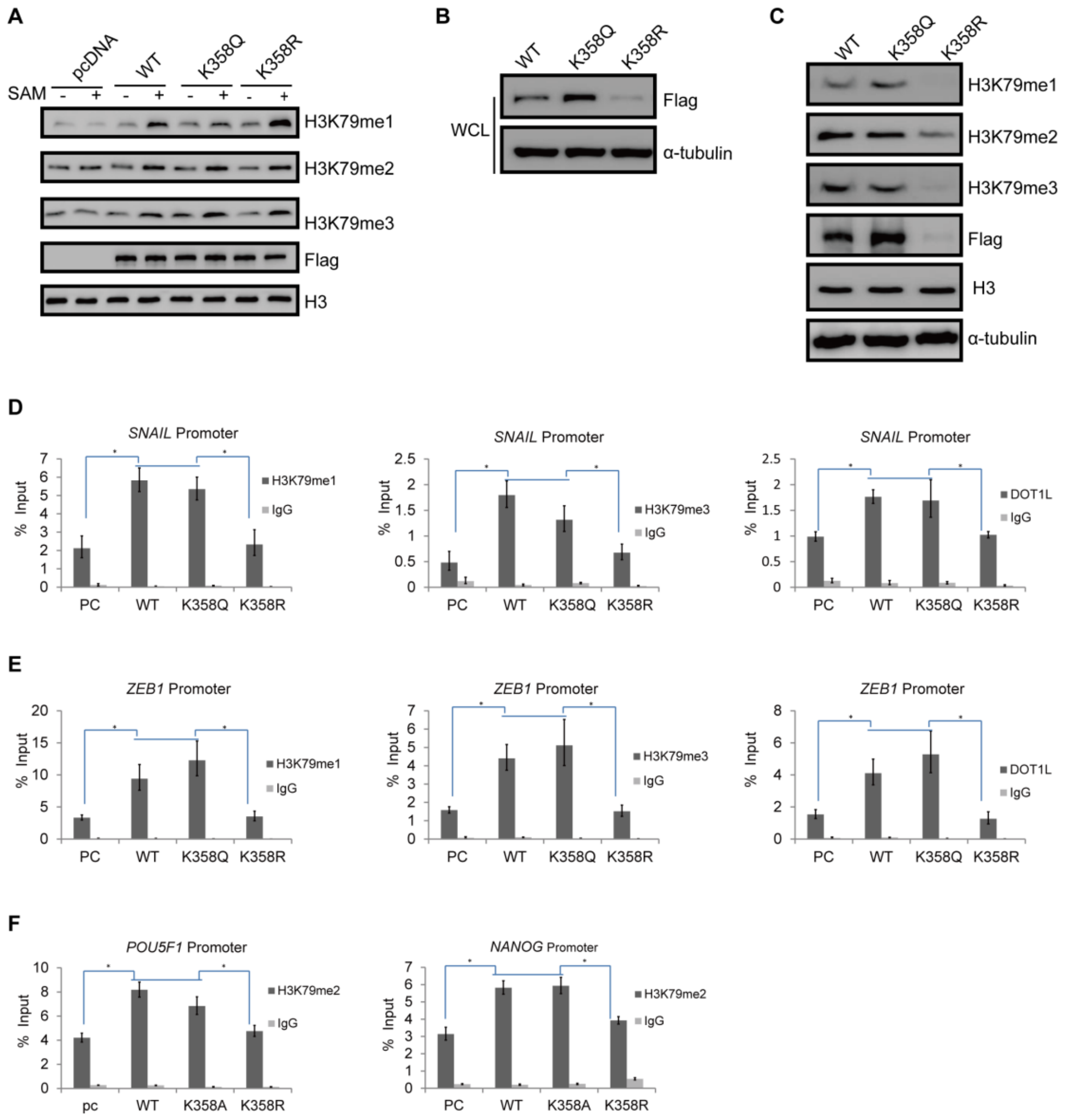

(A) HCT116 cells were transfected with pcDNA, Flag-DOT1L(WT), Flag-DOT1L(K358Q) or Flag-DOT1L(K358R), and the Flag immunoprecipitates were incubated with histones extracted from HCT116 cells separately. Western blotting was performed to detect H3K79me1/2/3 levels with the indicated antibodies. (B) Whole cell lysates (WCL) were extracted from LoVo cells transfected with Flag-DOT1L(WT), Flag-DOT1L(K358Q) or Flag-DOT1L(K358R), and were subjected to western blotting with the indicated antibodies. (C) WCL and histones were extracted from LoVo cells transfected with Flag-DOT1L(WT), Flag-DOT1L(K358Q) or Flag-DOT1L(K358R), and western blotting was performed to detect to H3K79 methylation with the indicated antibodies. (D, E) ChIP–qPCR analysis showing the amount of indicated proteins (H3K79me1, H3K79me3, DOT1L) recruited to the *SNAIL* (D) and *ZEB1* (E) promoter regions. The data represent the means $\pm$ SD (n=3). *p < 0.05. (F) H3K79me2 binding to the *Pou5F1* and *NANOG* promoter regions was analyzed by ChIP–qPCR. The data represent the means $\pm$ SD (n=3). *p < 0.05.

Figure S3 DOT1L acetylation regulates CRC migration, invasion and metastasis *in vivo*. Related to Figure 3.

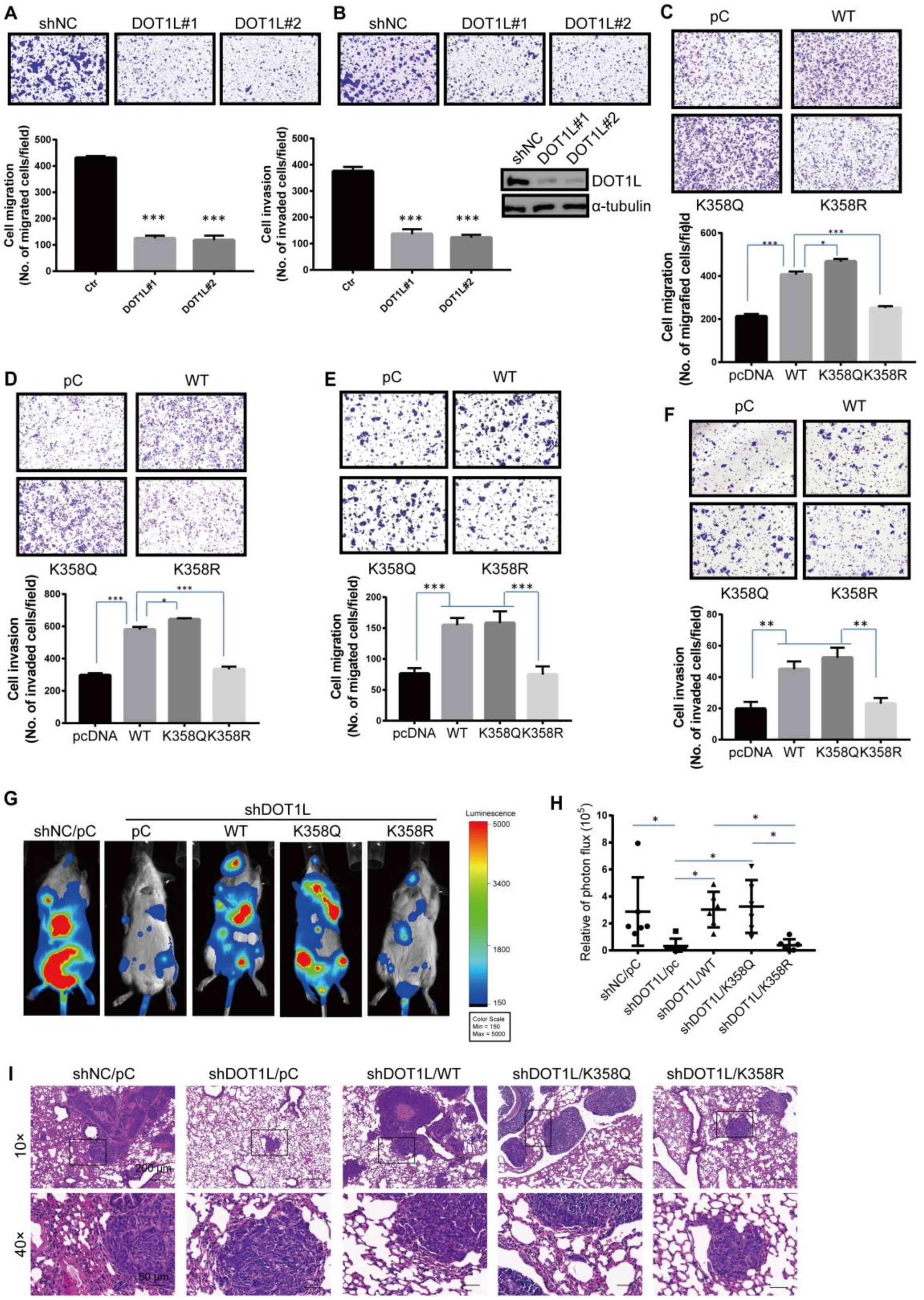

(A, B) Transwell cell migration (A) and matrigel cell invasion (B) assays were performed in DOT1L knockdown HCT116 cells (upper). The data represent the means ± SEM (n = 3) (lower). ***p < 0.001. The validated transfection of DOT1L specific siRNAs were detected by western blotting (lower). (C, D) Transwell cell migration (C) and matrigel cell invasion (D) assays in LoVo cells transfected with either pcDNA, DOT1L(WT), DOT1L(K358Q) or DOT1L(K358R) plasmids (upper). The data represent the means ± SEM (n = 3) (lower). *p < 0.05, ***p < 0.001. (E, F) Transwell cell migration (E) and matrigel cell invasion assays (F) in pcDNA-, DOT1L(WT)-, DOT1L(K358Q)- or DOT1L(K358R)-transfected SW480 cells (upper). The data represent the means ± SEM (n = 3) (lower). **p < 0.01, ***p < 0.001. (G, H) *In vivo* lung metastatic assays. Cells were injected into the tail veins of 6-week-old male NCG mice (n = 6 mice per group). Bioluminescence imaging was conducted 4 weeks after implantation. Representative *in vivo* bioluminescent images from the different groups (G) and the bioluminescent quantitation of lung metastases (H) are shown. *p < 0.05 compared with the shDOT1L/pC or shDOT1L/K358R. The data are presented as the means ± SD. (I) Representative lung metastasis specimens were sectioned and stained with H&E. Scale bars: 200 μm (upper); 50 μm (lower).

Figure S4 CBP mediates DOT1L acetylation *in vivo* and *in vitro* and confers DOT1L stability.

Related to Figure 4.

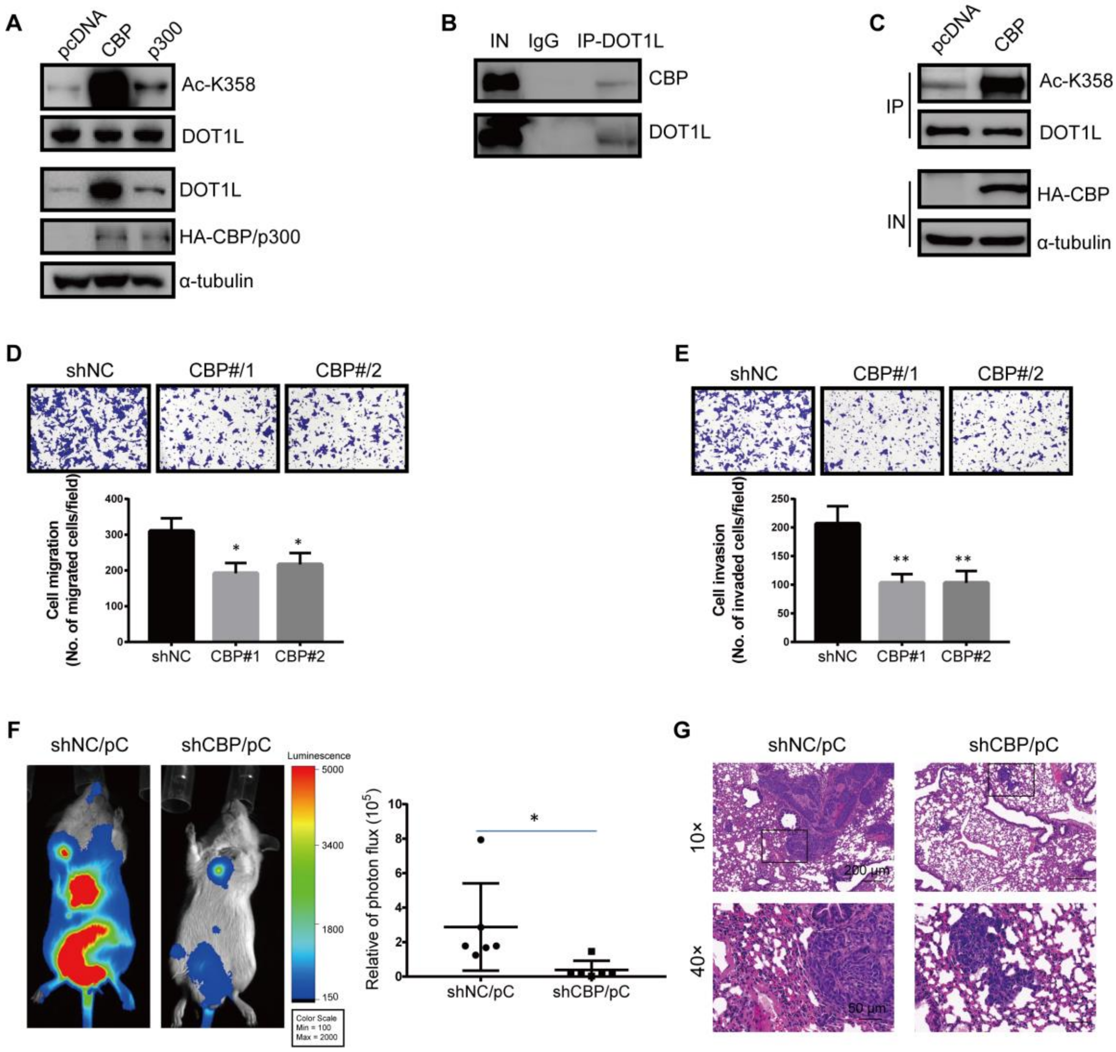

(A) LoVo cells were transfected with control pcDNA, HA-CBP or HA-p300, and DOT1L immunoprecipitates were analyzed by western blotting with the indicated antibodies. (B) Endogenous co-IPs to detect the interaction between DOT1L and CBP in LoVo cells. (C) LoVo cells were transfected with pcDNA or HA-CBP, and the DOT1L immunoprecipitates were analyzed by western blotting with the indicated antibodies. (D, E) Transwell cell migration (D) and matrigel cell invasion (E) assays were performed in HCT116 cells transfected with non-specific siRNA negative control shRNA (shNC) or CBP siRNA. The data represent the means ± SEM (n = 3). *p < 0.05, **p < 0.01. (F) *In vivo* lung metastatic assays. Cells were injected into the tail veins of 6-week-old male NCG mice (n = 6 mice per group). Bioluminescence imaging was conducted 4 weeks after implantation. Representative *in vivo* bioluminescent images from the different groups (left) and the bioluminescent quantitation of lung metastases (right) is shown. *p < 0.05 compared with the control (shNC/pC). The data are presented as the means ± SD. (G) Representative lung metastasis specimens were sectioned and stained with H&E. Scale bars: 200 μm (upper); 50 μm (lower).

Figure S5 E3 ligase RNF8 ubiquitinates DOT1L prior to degradation. Related to Figure 5.

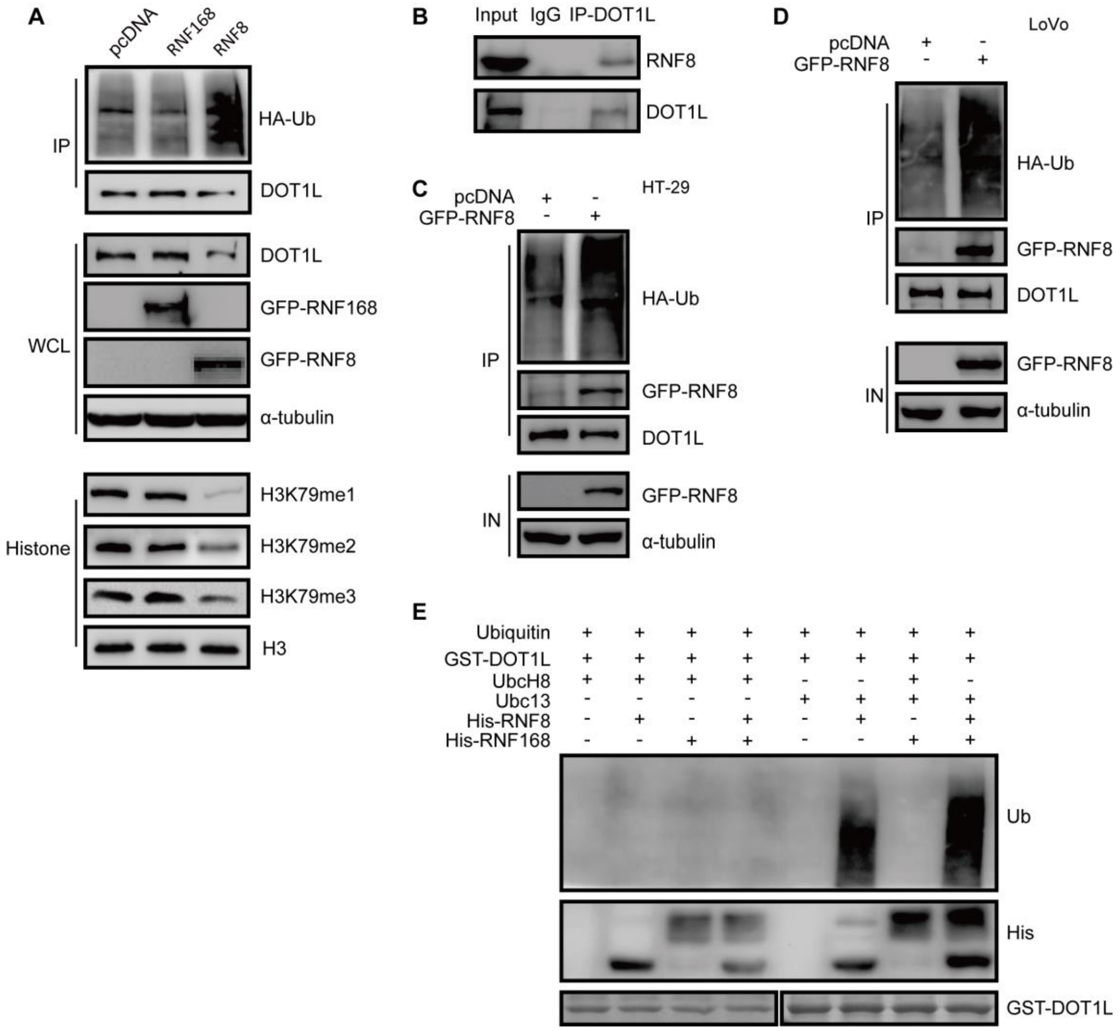

(A) LoVo cells were transfected with control pcDNA, GFP-RNF8 or GFP-RNF168 separately, and the cell lysates were subjected to western blotting with the indicated antibodies to detect DOT1L ubiquitination. (B) The endogenous CoIP was performed in LoVo cells with anti-RNF8 and anti-DOT1L antibodies. (C, D) HT-29 (C) and LoVo cells (D) were co-transfected with HA-Ub, pcDNA or GFP-RNF8, and DOT1L immunoprecipitates were analyzed by western blotting with the indicated antibodies. (E) *In vitro* ubiquitination assays were performed as follows: GST-DOT1L, UBE1 (E1), Ubc13 (E2) or UbcH8 (E2), His-RNF8 (E3), and His-RNF168 (E3) were incubated in specific buffer at 37 ℃ for 1 h before western blotting with the indicated antibodies.

Figure S6 DOT1L acetylation protects DOT1L from degradation by a RNF8–DOT1L interaction.

Related to Figure 6.

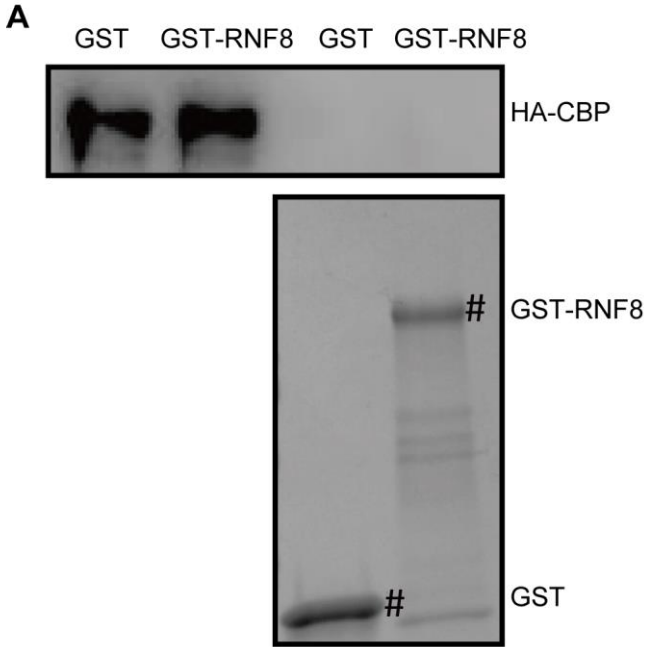

(A) Purified GST or GST-RNF8 was incubated with HA-CBP prior to western blotting to detect an interaction between HA-CBP and GST-RNF8.

Figure S7 DOT1L acetylation levels positively correlate with CBP expression and is associated with CRC metastasis and progression. Related to Figure 7.

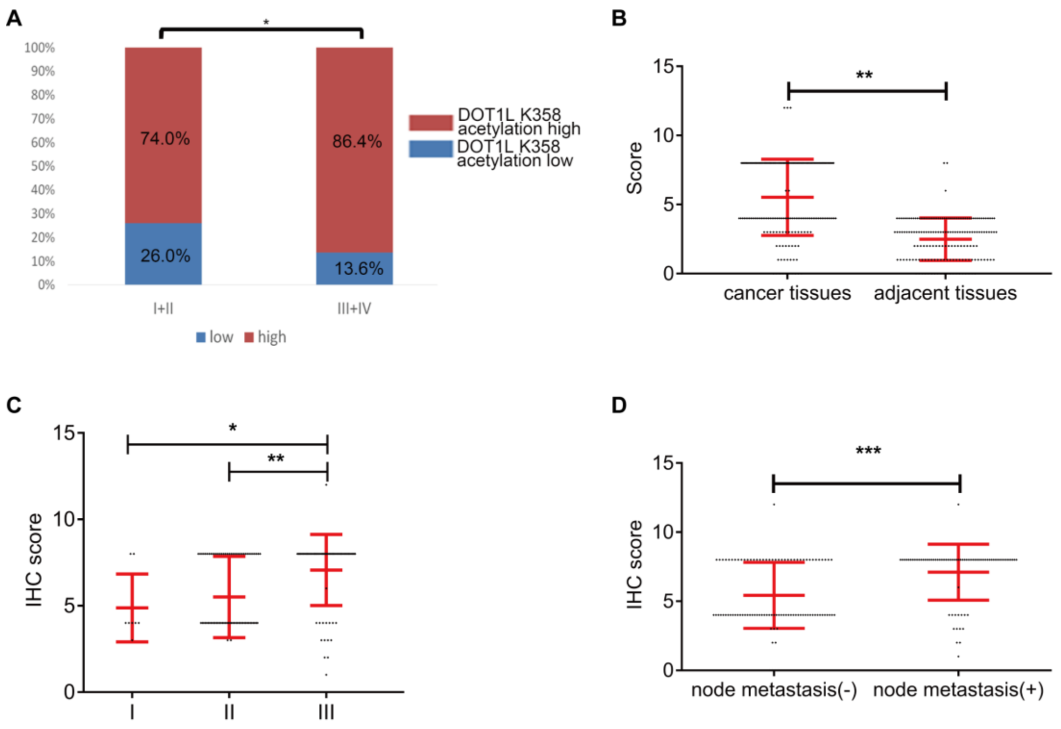

(A) Percentage of patients with high or low DOT1L(K358) acetylation levels according to tumour differentiation stage. The numbers in the bars represent the percentage of patients. The data were analyzed by Student's *t*-test; $*p < 0.05$. (B) The CBP score was compared between normal and CRC tissues and analyzed by paired Student's *t*-test; n =155; the data represent the means ± SD, error bars are shown in red; $**P < 0.01$. (C) CBP staining scores were determined by evaluating the extent and intensity of immune-positivity. The data were analyzed by two-tailed unpaired Student's *t*-test; the data represent the means ± SD, error bars are shown in red; $*p < 0.05$, $**P<0.01$. (D) The relative CBP levels were compared between colon adenocarcinoma tissues with and without lymph node metastasis. The data were analyzed by unpaired Student's *t*-test; the data represent the means ± SD, error bars are shown in red; $***P < 0.001$.

Table S1 DOT1L(K358) acetylation levels in cancerous and healthy colon tissues. Related to Figure 7.

	n	DOT1L(K358) acetylation levels		$\chi 2$	P value
		High(%)	Low(%)		
Colon carcinoma	155	125	30	18.592	0.000
Healthy colon tissues	155	90	65		

* Statistically significant ($p < 0.05$)

Table S2 Associations between DOT1L(K358) acetylation levels and key clinicopathological characteristics. Related to Figure 7.

	Variables	DOT1L(K358) acetylation levels		Total	χ2	P value
		Low	High			
Age (years)					0.184	0.668
	≤68	19	78	97		
	＞68	21	74	95		
	null					
T stage					3.312	0.069
	T1/T2	4	5	9		
	T3/T4	34	142	176		
TNM stage					4.398	0.036
	I/II	25	71	96		
	III/IV	12	76	88		
	null					
N stage					5.087	0.024
	N0	27	76	103		
	N1/N2	11	74	85		
	null					
M stage					0.013	0.91
	M0	38	146	184		
	M1	2	7	9		
Sex					0.001	0.976
	Female	17	65	82		
	Male	23	87	110		
	Null					
Grade					1.773	0.412
	I/II	15	66	81		
	III	23	72	95		

Table S3 Univariate and multivariate analyses of the factors correlated with overall survival in patients with CRC. Related to Figure 7.

variables	Univariate analysis			Multivariate analysis		
	HR	95%CI	P value	HR	95%CI	P value
DOT1L(K358) acetylation levels	2.526	1.384-4.610	0.003	2.796	0.967-8.083	0.058
Sex	1.021	0.693-1.502	0.918			
Grade	3.881	2.660-5.662	0.000	2.197	1.173-4.117	0.014
Age	1.449	0.984-2.134	0.060			
T stage	1.365	0.946-1.968	0.096			
N stage	2.073	1.571-2.736	0.000	1.217	0.557-2.661	0.622
M stage	3.477	1.737-6.963	0.000	0.436	0.085-2.250	0.322
TNM stage	2.042	1.509-2.764	0.000	1.468	0.567-3.801	0.430
MSH6	0.106	0.042-0.267	0.000	0.182	0.059-0.559	0.003
MSH2	0.212	0.120-0.372	0.000	0.477	0.246-0.924	0.028
MLH1	0.975	0.557-1.705	0.928			
KI67	0.745	0.426-1.304	0.302			
P53	0.975	0.572-1.664	0.927			

* Statistically significant (p<0.05)

Table S4 CBP expression level in cancerous and healthy colon tissues. Related to Figure 7.

	n	CBP expression level		χ2	P value
		High(%)	Low(%)		
Colon carcinoma	154	68	86	65.583	0.000
Healthy colon tissues	154	7	147		

* Statistically significant ($p < 0.05$)

Table S5 Associations between CBP expression level and CRC clinicopathological characteristics. Related to Figure 7.

	Variables	CBP expression level		Total	χ2	P value
		Low	High			
Age (years)					0.137	0.711
	≤68	50	45	95		
	＞68	52	42	94		
	null					
T stage					0.575	0.448
	T1/T2	6	3	9		
	T3/T4	93	80	173		
TNM stage					4.53	0.033
	I/II	57	37	94		
	III/IV	39	48	87		
	null					
N stage					5.54	0.019
	N0	62	39	101		
	N1/N2	37	47	84		
M stage					0.007	0.934
	M0	98	83	181		
	M1	5	4	9		
	null					
Sex					0.291	0.59
	Female	45	35	80		
	Male	57	52	109		
	Null					
Grade					0.22	0.639
	I	9	6	15		
	II/III	94	81	175		

Table S6 Univariate and multivariate analyses of the factors correlated with overall survival in patients with CRC. Related to Figure 7.

variables	Univariate analysis			Multivariate analysis		
	HR	95%CI	P value	HR	95%CI	P value
CBP expression	1.684	1.145-2.475	0.008	0.913	0.443-1.882	0.805
sex	0.975	0.662-1.438	0.899			
Grade	3.848	2.630-5.630	0.000	2.021	1.053-3.880	0.035
Age	1.475	0.999-2.177	0.051			
T stage	1.353	0.939-1.950	0.105			
N stage	2.034	1.539-2.689	0.000	1.361	0.625-2.963	0.438
M stage	3.454	1.724-6.920	0.000	0.470	0.090-2.464	0.372
TNM stage	1.998	1.475-2.707	0.000	1.537	0.555-4.254	0.408
MSH6	0.101	0.040-0.257	0.000	0.167	0.054-0.515	0.002
MSH2	0.216	0.123-0.381	0.000	0.456	0.234-0.889	0.021
MLH1	0.933	0.533-1.632	0.808			
KI67	0.723	0.413-1.264	0.255			
P53	0.943	0.553-1.608	0.829			

* Statistically significant ($p<0.05$)
